# Gold nanoclusters eliminate obesity induced by antipsychotics

**DOI:** 10.1038/s41598-022-09541-x

**Published:** 2022-04-01

**Authors:** Meng He, Jing Yao, Zijun Zhang, Ying Zhang, Rui Chen, Zhenhua Gu, XuFeng Huang, Chao Deng, Ruqin Zhou, Jun Fan, Baohua Zhang, Yanqian Xie, Guanbin Gao, Taolei Sun

**Affiliations:** 1 School of Chemistry, Chemical Engineering and Life Sciences, Wuhan University of Technology, Wuhan, 430070 China; 2 State Key Laboratory of Advanced Technology for Materials Synthesis and Processing, Wuhan University of Technology, Wuhan, 430070 China; 3 School of Medicine and Molecular Horizons, University of Wollongong, Wollongong, NSW 2522 Australia; 4 The National Clinical Research Center for Mental Disorders and Beijing Key Laboratory of Mental Disorders, Capital Medical University, Beijing, 100191 China

**Keywords:** Endocrine system and metabolic diseases, Translational research, Nanoparticles

## Abstract

Obesity induced by antipsychotics have plagued more than 20 million people worldwide. However, no drug is available to eliminate the obesity induced by antipsychotics. Here we examined the effect and potential mechanisms of a gold nanoclusters (AuNCs) modified by *N*-isobutyryl-*L*-cysteine on the obesity induced by olanzapine, the most prescribed but obesogenic antipsychotics, in a rat model. Our results showed that AuNCs completely prevented and reversed the obesity induced by olanzapine and improved glucose metabolism profile in rats. Further mechanism investigations revealed that AuNCs exert its anti-obesity function through inhibition of olanzapine-induced dysfunction of histamine H1 receptor and proopiomelanocortin signaling therefore reducing hyperphagia, and reversing olanzapine-induced inhibition of uncoupling-protein-1 signaling which increases thermogenesis. Together with AuNCs’ good biocompatibility, these findings not only provide AuNCs as a promising nanodrug candidate for treating obesity induced by antipsychotics, but also open an avenue for the potential application of AuNCs-based nanodrugs in treating general obesity.

## Introduction

Obesity have been becoming a substantial and growing burden on public health. In particularly, obesity induced by antipsychotics have been affected more than 20 million patients globally^1–3^. It has been reported that the risk for antipsychotics-treated patients to develop obesity is 3–4 times higher than the general population^4,5^. Among the antipsychotics, olanzapine is the most efficacious and prescribed second-generation antipsychotic medication^6,7^. Olanzapine is also one of the most obesogenic drugs^8,9^. Clinical reports have shown that up to 86% of patients who took olanzapine experienced weight gain or obesity^6,10,11^. The obesity caused by olanzapine has been associated with induction of dyslipidemia and hyperglycemia, increase of the risk of cardiovascular disease and stroke, and increase of mortality rates of schizophrenia patients^12,13^. Moreover, olanzapine has been implicated in glucose metabolic dysfunction^14,15^ and significantly increased risk of developing type-II diabetes in patients^16,17^. Last two decades have witnessed great efforts in development of drugs addressing weight gain/obesity induced by olanzapine and other antipsychotics^18–20^. Recent studies have reported that existing anti-obesity and anti-diabetic medications could provide limited relief for the obesity caused by olanzapine^21,22^. However, there is still lack of effective medication to complete prevent and/or reverse the obesity induced by olanzapine.

Energy homeostasis is tightly controlled by the hypothalamus, which receives and integrates neural, metabolic, and humoral signals to regulate food intake and body weight^23^. In the hypothalamus, histamine neurons are widely distributed and suppress food intake via activating the histamine H1 receptors (H1Rs)^24^. The proopiomelanocortin (POMC) neurons, which are found in the arcuate nucleus (Arc) of the hypothalamus, are also involved in food intake and body weight regulation^25^. Evidence suggests that the H1Rs and POMC neurons in the hypothalamus significantly contribute to olanzapine-induced obesity. Olanzapine stimulated AMP-activated protein kinase (AMPK) signaling by blocking the hypothalamic H1Rs, resulting in hyperphagia and weight gain^26,27^. Furthermore, olanzapine treatment largely lowered the hypothalamic POMC mRNA and protein expression, contributing to hyperphagia and obesity^28,29^. In addition to the hypothalamus, reduced thermogenesis in brown adipose tissue (BAT) plays a key role in olanzapine-induced obesity. Olanzapine treatment reduced BAT temperature and decreased the expression of biomarkers for BAT thermogenesis including uncoupling protein-1 (UCP-1) and peroxisome proliferator-activated receptor-gamma coactivator-1α (PGC-1α)^30^. These evidences suggested that potential drug candidates which could regulate the hypothalamic H1R-AMPK signaling, POMC expression and BAT thermogenesis might significantly prevent olanzapine-induced obesity.

Gold nanoparticles are small gold particles with a diameter of 1–100 nm^31^. Emerging data suggested that gold nanoparticles hold great potential for biomedical application as anti-obese drug. It has been reported that gold nanoparticles effectively prevented and treated high-fat diet induced obesity and reversed the corresponding glucose metabolic disorder in rodents^32,33^. Gold nanoclusters (AuNCs), an ultra-small gold nanoparticle with dimeter small than 3 nm, have been widely studied in bioimaging^34^, bioassays^35,36^ and treatments of diseases^37–39^, etc. in last decade. Recently, AuNCs have drawn great attention in the development of nanomedicine because of their unique physicochemical property, good bioactivity and biocompatibility^40,41^. Herein, we studied the curative effects of a AuNCs modified by *N*-isobutyryl-l-cysteine (NIBC) on the obesity induced by olanzapine and their underlying molecular mechanisms in a rat model.

## Results

### Preparation and characterization of AuNCs

**Figure 1:**
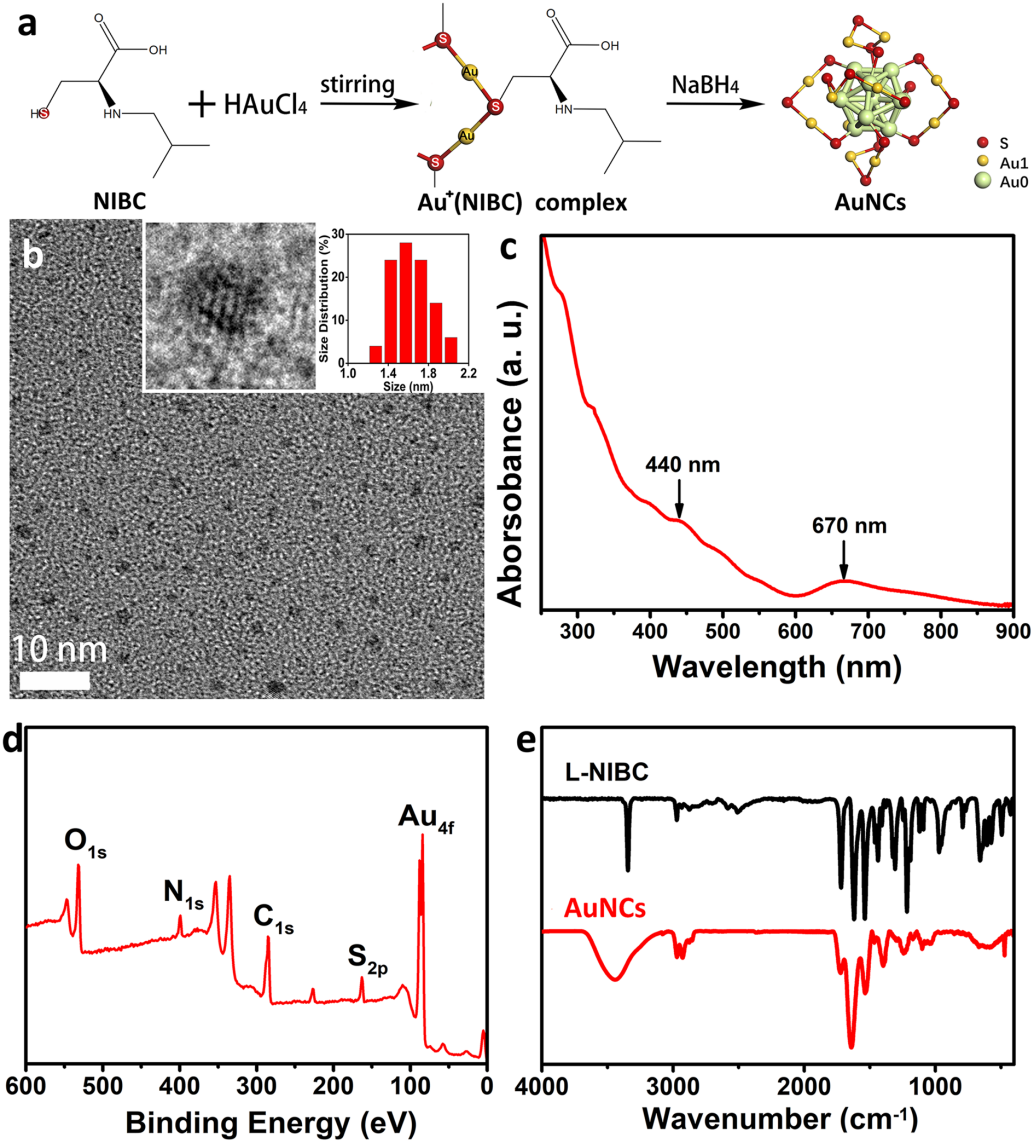
Scheme of synthesizing and characterization of AuNCs. (**a**) Diagrammatic flow chart showing the scheme of synthesizing of AuNCs. (**b**) TEM images, (**c**) UV–Vis–NIR absorption spectrum and (**d**) XPS survey spectrum of AuNCs. (**e**) FT-IR spectra of AuNCs and NIBC molecules.

In this study, AuNCs modified by NIBC with a diameter smaller than 2 nm was synthesized in one-pot using a two-step method (Fig. 1a). First, HAuCl_4_ and NIBC were incubated in a cool bath with a gently stirring to obtain react intermediates of Au^+^(NIBC) complex. Second, NaBH_4_ was added into the mixtures and incubated with a strongly stirring at room temperature to produce the final products of AuNCs. After dialysis and lyophilization, pure AuNCs were prepared, and then characterized by TEM, UV–Vis–NIR absorption spectroscopy, XPS and fourier transform infrared spectroscopy (FT-IR). The TEM images of AuNCs (Fig. 1b) presented spherical particles with high dispersibility. The statistic diameter of particles was 1.6 ± 0.5 nm (right inset of Fig. 1b). The HR-TEM image of AuNCs (left inset of Fig. 1b) exhibited clear lattice fringes. The UV–vis spectrum of AuNCs in water (Fig. 1c) showed two absorption peaks around 440 and 670 nm, which is the typical spectroscopic characteristics of Au_25_(SR)_18_ according to the previous report^42^. In the XPS survey spectrum of AuNCs (Fig. 1d), the characteristic peaks of C1s, O1s, N1s, S2p and Au4f could be found, indicating that the NIBC molecules were successfully modified onto the surfaces of AuNCs. The FT-IR spectra of AuNCs and free NIBC molecules were shown in Fig. 1e. Compared with free NIBC molecules, the disappearance of the stretching vibration peak (*ν* = 2566 cm^−1^) of –S–H indicated that NIBC molecules were successfully grafted to AuNCs via Au–S bonds. All the above characterization demonstrated that AuNCs has been successfully prepared.

### AuNCs completely prevents and reverses olanzapine-induced obesity in rats

Olanzapine-induced obesity was largely attributed to its antagonistic effect on H1Rs and thus regulated the downstream AMPK signaling^27,43,44^. These molecules were not related to the main therapeutic effect of olanzapine^45^. Our preliminary studies have investigated the effect of AuNCs on the H1R-AMPK signaling in cells. By using in vitro immunostaining analysis, we found that olanzapine increased H1R and phosphorylated AMPK (pAMPK) fluorescence intensity (Fig. S1a–c), while co-treatment with AuNCs high dose (20 mg/L) significantly suppressed the olanzapine-induced increase in H1R and pAMPK fluorescence intensity after both 2 h and 24 h olanzapine treatment (all *p* < 0.05). Co-treatment of AuNCs at a low dose (10 mg/L) suppressed H1R fluorescence intensity (*p* < 0.05) but not pAMPK fluorescence intensity after 2 h treatment. After 24 h treatment, co-treatment of AuNCs low dose did not significantly inhibit olanzapine-induced increase in H1R and pAMPK fluorescence intensity in SH-SY5Y cells (Fig. S1). These results encouraged us to further study the in vivo effects of AuNCs on obesity induced by olanzapine.

**Figure 2:**
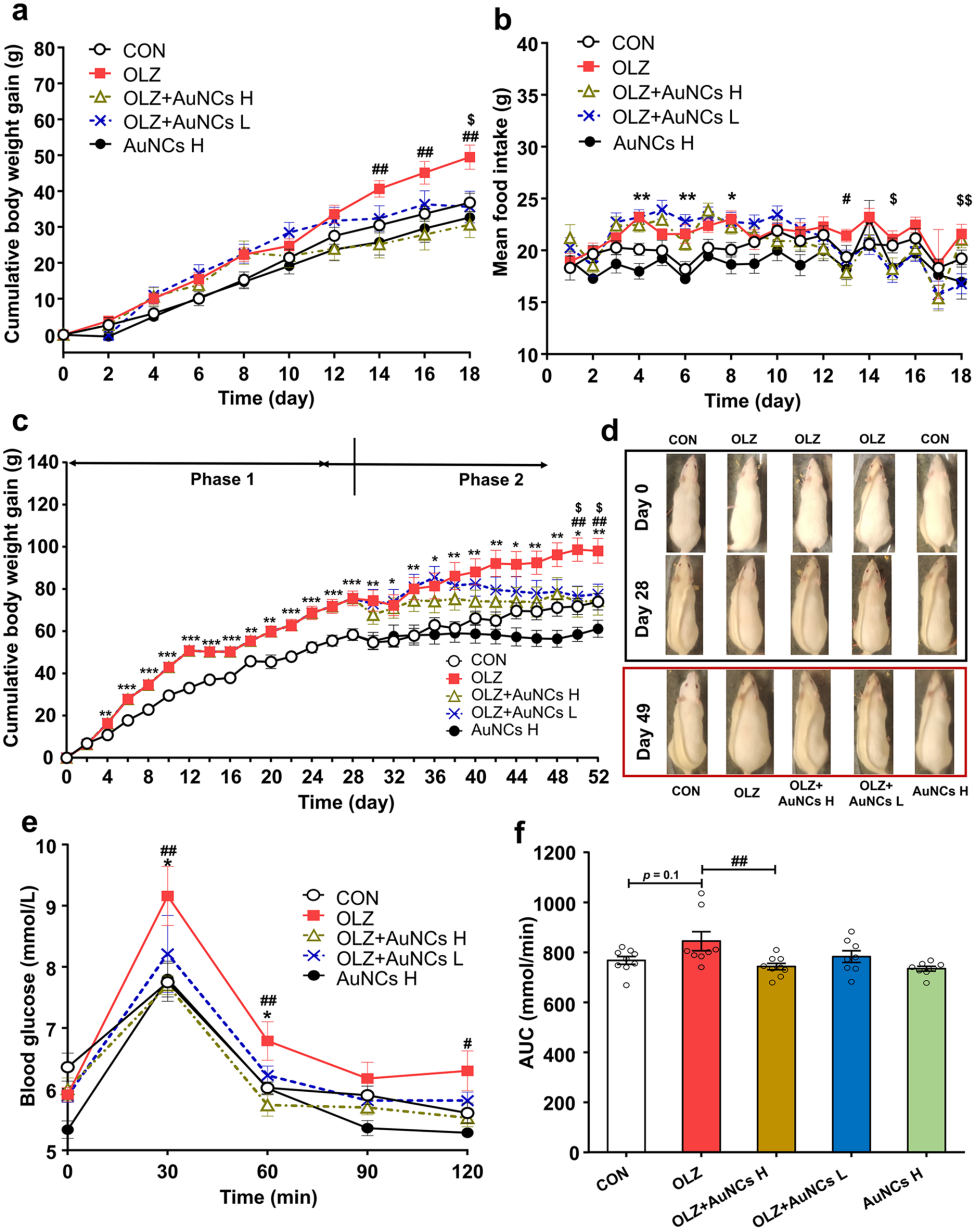
Effects of AuNCs on preventing and reversing the obesity induced by olanzapine and ameliorating glucose metabolic disorder caused by olanzapine. (**a**,**b**) Preventive effect of AuNCs on olanzapine-induced weight gain and food intake in rats. (**c**,**d**) Effects of AuNCs on established obesity induced by olanzapine administration in rats. (**e**,**f**) Effects of AuNCs on olanzapine-induced glucose metabolism examined by an intraperitoneal glucose tolerance test (IPGTT) in rats. All data were presented as mean ± SEM, *n* = 9–14/group. *p < 0.05, **p < 0.01, ***p < 0.0001, OLZ vs. CON; ^#^*p* < 0.05, ^##^*p* < 0.01, OLZ + AuNCs H vs. OLZ; ^$^*p* < 0.05, ^$$^*p* < 0.01, OLZ + AuNCs L vs. OLZ.

The effects of AuNCs on preventing or reversing olanzapine-induced weight gain were investigated in a rat model. As shown in Fig. 2a, olanzapine treatment induced weight gain while AuNCs high dose co-treatment effectively reduced olanzapine-induced weight gain from the 10th day of administration and this effect reached significantly statistical difference from 14th day of administration (14th day: *p* = 0.003; 16th day: *p* = 0.004; 18th day: *p* = 0.002). The weight gain of rats in OLZ + AuNCs H group was similar to that of the CON group during 10th–18th day of treatment. AuNCs low dose co-treatment started to reduce olanzapine-induced weight gain on 12th day of administration and this effect reached significant difference on 18th day of administration (*p* = 0.025) (Fig. 2a). The results suggested that AuNCs suppressed olanzapine-induced weight gain in a dose- and time-dependent manner. Moreover, AuNCs high dose co-treatment decreased olanzapine-induced hyperphagia which was significant on 13th day of co-treatment (*p* = 0.041) (Fig. 2b). AuNCs low dose reduced hyperphagia on 15th (*p* = 0.022) and 18th day (*p* = 0.005) of co-treatment. Furthermore, olanzapine treatment caused increased peripheral mesenteric fat (*p* = 0.036), which is a significant marker of obesity^46^. This effect was inhibited by both AuNCs high dose (29.09 ± 0.16%, *p* = 0.003) and low dose co-treatment (21.47 ± 0.11%, *p* = 0.032) (Fig. S2). The above results demonstrated that AuNCs completely prevented olanzapine-induced weight gain/obesity.

In clinic, numerous patients who have been prescribed with antipsychotics for long periods have already been overweight. Therefore, we further investigated whether AuNCs could reverse the obesity induced by olanzapine by using a rat model that was already obese due to chronic olanzapine administration. We have established a chronic olanzapine-induced obese rat model by treating rats with olanzapine for 28 days according to previous studies (marked as phase 1)^30,43^. After the rats were significantly obese, rats were co-treated olanzapine with AuNCs high dose and low dose for another 28 days (marked as phase 2). As shown in Fig. 2c,d, in phase 1, olanzapine-only treatment significantly induced weight gain (all *p* < 0.001). In phase 2, olanzapine-treated rats continued gaining weight and became extremely obese at the end of treatment (all *p* < 0.05). AuNCs high dose co-treatment significantly prevented olanzapine-treated rats from obesity. Indeed, from day 34th–52nd, OLZ + AuNCs H experimental group did not gain weight. AuNCs low dose co-treatment started to reduce olanzapine-induced weight gain on day 38th and this effect reached a significant difference on day 50th–52nd. Moreover, at the end of co-treatment, both AuNCs high dose and low dose co-treated groups had similar weight gain compared with control (original figures of rats were shown in Fig. S7 from S7-1 to S7-15). These results demonstrated that AuNCs completely reversed the established obesity induced by olanzapine.

### AuNCs prevents olanzapine-induced increases in blood glucose

Patients treated with obesogenic antipsychotic are usually associated with glucose metabolic disorder and more likely to develop type-II diabetes^8^. The effects of olanzapine and AuNCs on glucose metabolism were examined by using an intraperitoneal glucose tolerance test (IPGTT). As shown in Fig. 2e, when rats were IP injected with 1 g/kg glucose, blood glucose concentration of olanzapine-treated rats rose rapidly at 30th min (*p* = 0.017) and 60th min (*p* = 0.015) (Fig. 2e) compared with that of the control rats. AuNCs high dose co-treatment largely reduced blood glucose concentration at 30th min (*p* = 0.007), 60th min (*p* = 0.003) and 120th min (*p* = 0.044) compared with olanzapine-only treatment (Fig. 2e). Olanzapine treatment also tended to increase the area under curve (AUC) of glucose (*p* = 0.1) compared with control (Fig. 2f). AuNCs high dose co-treatment significantly decreased AUC of glucose compared with olanzapine-only treatment (*p* = 0.007) (Fig. 2f). AuNCs low dose co-treatment had no significant inhibitory effect on blood glucose concentration. The effect of olanzapine and AuNCs treatment on plasma hormone and lipid metabolism was also examined. Compared with control, olanzapine and AuNCs treatment did not obviously affect cholesterol, triglyceride, leptin and insulin levels in plasma (*p* > 0.05, Fig. S3, S4).

### AuNCs inhibits olanzapine-induced hypothalamic H1R-AMPK signaling dysfunction and increases POMC expression

**Figure 3:**
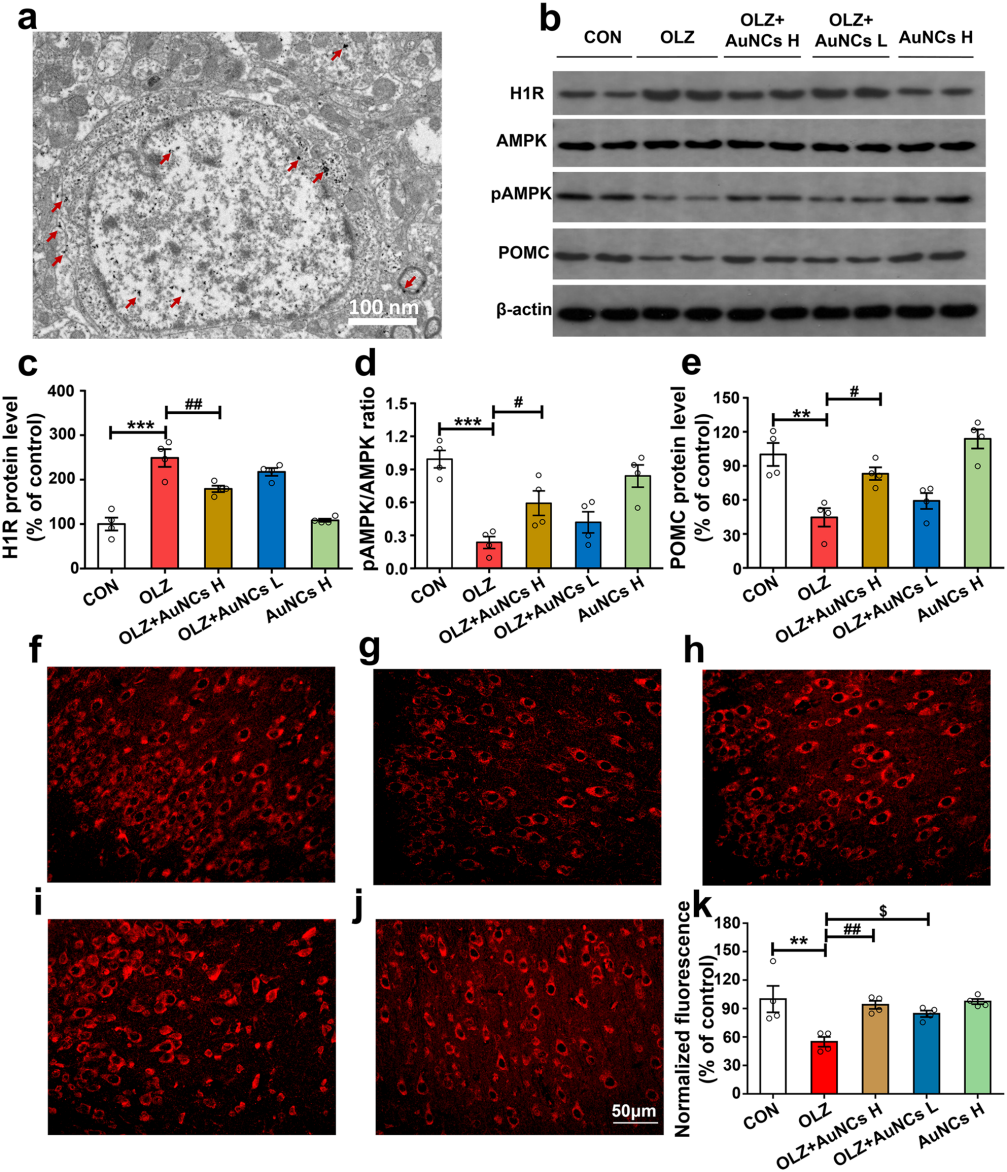
Effects of olanzapine and AuNCs co-treatment on H1R-AMPK signaling, POMC protein expression and POMC immunofluorescence staining in the hypothalamus. (**a**) TEM image of a hypothalamic slice at 6th h after IP injection of 20 mg/kg AuNCs in rats. The presence of AuNCs was marked by red arrows. (**b**) Representative western blot figures of H1R, AMPK, pAMPK and POMC in the hypothalamus after co-treatment of olanzapine and AuNCs. (**c**–**e**) Densitometry analysis of H1R expression (**c**), pAMPK/AMPK (**d**) and POMC expression (**e**). (**f**–**k**) POMC immunofluorescence staining in the hypothalamic Arc of rats in CON (**f**), OLZ (**g**), O + AuNCs H (**h**), O + AuNCs L (**i**), AuNCs H (**j**) group and the corresponding quantification of POMC fluorescence intensity (**k**). *n* = 4/group. All data were presented as mean ± SEM. *p < 0.05, **p < 0.01, ***p < 0.0001, OLZ vs. CON; ^#^*p* < 0.05, ^##^*p* < 0.01, ^###^*p* < 0.0001, OLZ + AuNCs H vs*.* OLZ; ^$^*p* < 0.05, OLZ + AuNCs L v*s.* OLZ. Original figures were shown in Fig. S8.

In vivo, the hypothalamic H1R-AMPK signaling has been identified as a key modulator in regulating food intake and body weight^27^. Therefore, we firstly examined whether AuNCs could reach the hypothalamus and mediate the hypothalamic H1R-AMPK signaling. As shown in Fig. 3a, by using Cryo-TEM, we have found that AuNCs existed in the hypothalamic cells of rat brain slices after being IP injected AuNCs with a dosage of 20 mg/kg for 2 h. This result demonstrated that AuNCs might directly act on hypothalamus to regulate the H1R-AMPK signaling in vivo, which may thus inhibit olanzapine-induced weigh gain. Therefore, we have further investigated the effect of AuNCs co-treatment on the H1Rs and AMPK signaling in the rat hypothalamus by using western blot. As shown in Fig. 3b,c, olanzapine-only treatment significantly up-regulated the hypothalamic H1R protein expression (*p* < 0.0001). AuNCs high dose but not low dose co-treatment significantly inhibited the H1R protein expression compared with olanzapine-only treatment (*p* = 0.004, Fig. 3c). H1R protein level was positively correlated with the cumulative food intake of rats (*r* = 0.71, *p* = 0.001). In this study, pAMPK/AMPK ratio in hypothalamus was used to better reflect AMPK activity in vivo based on previous work^28^. Different with the in vitro study, the hypothalamic pAMPK/AMPK ratio in OLZ + AuNCs H group was significantly decreased compared with that of OLZ group (*p* < 0.0001, Fig. 3d). We have also found that AuNCs high dose but not low dose co-treatment significantly reversed olanzapine-induced decrease in pAMPK/AMPK ratio (*p* = 0.0046, Fig. 3d) (Original figures were shown in Fig. S8). The pAMPK/AMPK ratio tended to negatively correlate with weight gain (*r* = − 0.41, *p* = 0.075) and food intake (*r* = − 0.41, *p* = 0.078). The above results suggested that AuNCs co-treatment suppressed olanzapine-induced H1R overexpression and AMPK signaling dysfunction in the hypothalamus, which resulted in decreased weight gain of AuNCs co-treated rats.

The hypothalamic POMC, producing many anorexigenic peptides, plays a key role in regulating feeding and body weight. This study found that olanzapine treatment largely decreased POMC protein expression in the hypothalamus compared with control (*p* = 0.001, Fig. 3b,e), consisting with previous studies^47,48^. AuNCs high dose co-treatment significantly reversed olanzapine-induced decrease in POMC protein expression (*p* = 0.013, Fig. 3e). The hypothalamic POMC protein level was negatively correlated with food intake (*r* = − 0.60, *p* = 0.008) (original figures were shown in Fig. S8). POMC cells in the hypothalamic Arc were also evaluated by using immunofluorescence staining. As shown in Fig. 3f–k, olanzapine treatment significantly decreased POMC fluorescence intensity compared with control (*p* = 0.002). Co-treatment with AuNCs high dose and low dose significantly reversed the olanzapine-induced decrease in POMC fluorescence intensity (high dose, *p* = 0.006; low dose, *p* = 0.036, respectively). These results indicated that AuNCs could also inhibit olanzapine-induced hyperphagia and weight gain through increasing the hypothalamic POMC expression.

### AuNCs reverses olanzapine-induced decreases in BAT thermogenesis markers

**Figure 4:**
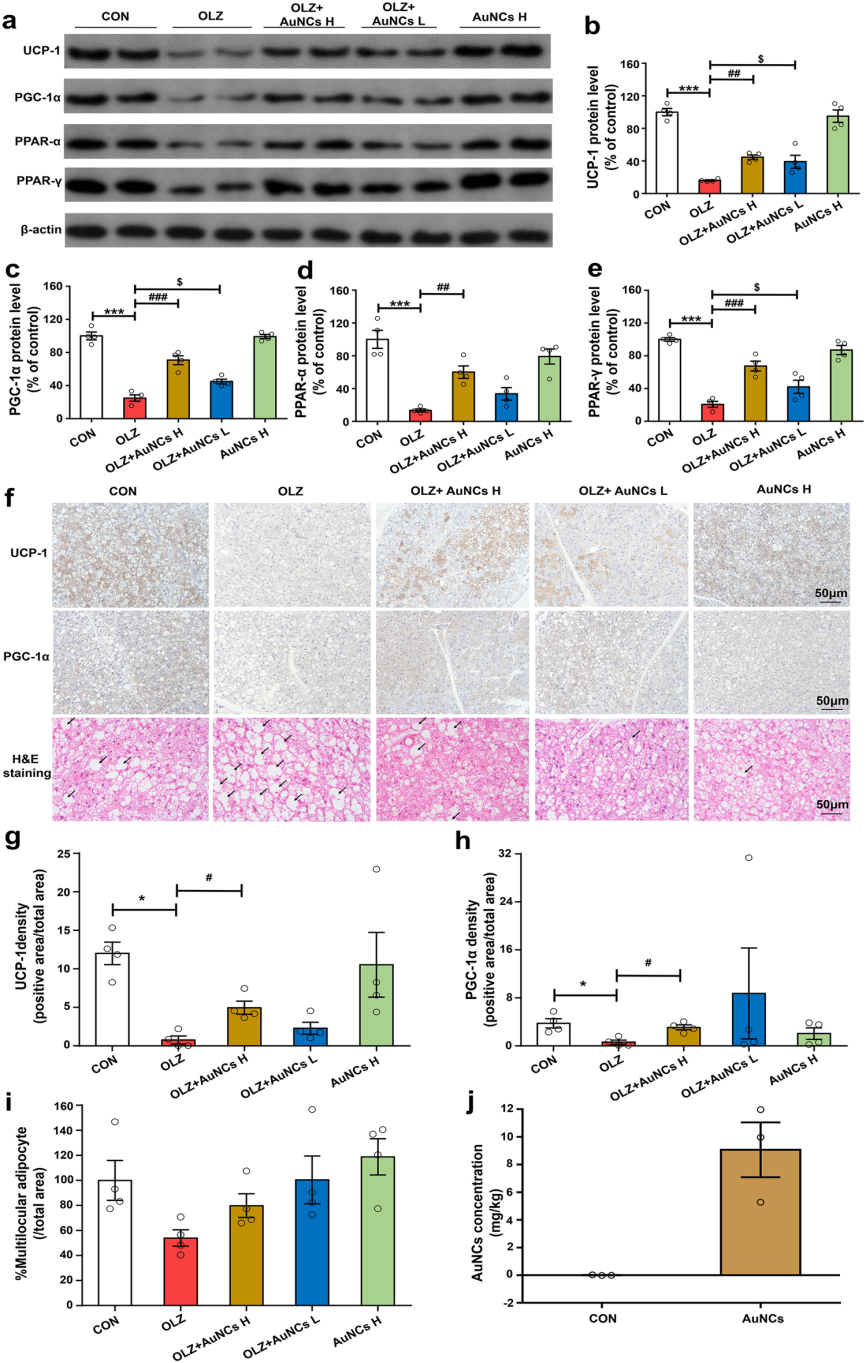
Effects of olanzapine and AuNCs co-treatment on the protein expression of BAT thermogenic markers. (**a**) Representative western blot figures of UCP-1, PGC-1α, PPAR-α, PPAR-γ protein expression in BAT. (**b**–**e**) Densitometry analysis of the protein expression of UCP-1 (**b**), PGC-1α (**c**), PPAR-α (**d**) and PPAR-γ (**e**). (**f**) Immunohistochemistry of UCP-1 and PGC-1α and H&E staining of BAT. (**g**–**i**) Quantification of relative positive area for UCP-1 (**g**), PGC-1α (**h**) and multilocular brown adipocytes (**i**). *n* = 4/group. (**j**) Concentrations of AuNCs in BAT of rats after administration of saline or 20 mg/kg AuNCs for 2 h. *n* = 3/group. All data were presented as mean ± SEM. *p < 0.05, ***p < 0.0001, OLZ vs*.* CON; ^#^*p* < 0.05, ^##^*p* < 0.01, ^###^*p* < 0.0001, OLZ + AuNCs H vs*.* OLZ; ^$^*p* < 0.05, OLZ + AuNCs L vs*.* OLZ. Original figures were shown in Fig. S9.

BAT, an energy-expending organ which produces heat, also plays an important role in obesity development. Inhibition of BAT thermogenesis induces obesity. In addition, BAT thermogenesis is controlled by the hypothalamus. The hypothalamus coordinates outflow signals that drive sympathetic activity to the BAT, controlling thermogenesis (hypothalamus-BAT axis)^49^. In the hypothalamus, the POMC neurons have been reported to be closely related to BAT thermogenesis^50^. Increased expression of POMC in the hypothalamus induced BAT thermogenesis in rats^51^. In this study, we have found that AuNCs significantly reversed olanzapine-induced decreases in POMC expression in the hypothalamus, suggesting that AuNCs might also regulate the BAT thermogenesis. Therefore, we further investigated the effect of AuNCs on BAT thermogenesis. In BAT, UCP-1 and PGC-1α are well known biomarkers for thermogenesis^52,53^. Peroxisome proliferators-activated receptors (PPAR-α and PPAR-γ), working as transcription factors, upregulate the expression of UCP-1 and PGC-1α, thus increase BAT thermogenesis^54–56^. It has been reported that olanzapine decreased BAT thermogenesis^30^. In this study, western blot analysis showed that olanzapine treatment dramatically downregulated the BAT thermogenic markers including UCP-1, PGC-1α, PPAR-α and PPAR-γ protein expression from 100% to 16.07 ± 0.82% (*p* < 0.0001), 24.91 ± 3.81% (*p* < 0.0001), 13.42 ± 1.84% (*p* < 0.0001) and 20.65 ± 3.63% (*p* < 0.0001) respectively compared with control (marked as 100%) (Fig. 4a–e). AuNCs high dose co-treatment significantly reversed the reduction of UCP-1 (*p* = 0. 007), PGC-1α (*p* < 0.0001), PPAR-α (*p* = 0. 003) and PPAR-γ (*p* < 0.0001) expression caused by olanzapine. AuNCs low dose co-treatment also significantly reversed UCP-1 (*p* = 0.030), PGC-1α (*p* = 0.011) and PPAR-γ (*p* = 0.046) but not PPAR-α expression compared with olanzapine-only treatment (Fig. 4a–e) (original figures were shown in Fig. S9 from S9-1 to S9-5). These results were confirmed by immunohistochemistry analysis which showed that olanzapine-only treatment largely decreased UCP-1 (*p* = 0.021) and PGC-1α (*p* = 0.021) density compared with control (Fig. 4f–h). AuNCs high dose co-treatment significantly increased UCP-1 (*p* = 0.021) and PGC-1α density (*p* = 0.021) compared with olanzapine-only treatment. AuNCs low dose co-treatment non-significantly increased UCP-1 (*p* > 0.05) and PGC-1α density (*p* > 0.05) compared with olanzapine-only treatment (Fig. 4f–h) (original figures were shown in Fig S9 from S9-6 to S9-15). Pearson correlation analysis showed that the protein expression of PGC-1α (*r* = − 0.46, *p* = 0.04) and PPAR-γ (*r* = − 0.50, *p* = 0.03) were negatively correlated with weight gain of rats. Furthermore, brown to white transdifferentiation, which was regulated by PGC-1α and UCP-1, is another important contributor to decreased BAT thermogenesis and obesity pathology. In this study, hematoxylin–eosin (H&E) staining (Fig. 4f) and quantitative analysis (Fig. 4g–i) revealed that in BAT, olanzapine slightly reduced the percentage of multilocular brown adipocytes (*p* = 0.1). AuNCs high does and low dose co-treatment non-significantly reversed these effects (*p* > 0. 05) (Original H&E staining figures were shown in Fig. S9 from S9-16 to S9-20). These results demonstrated that AuNCs could reverse the olanzapine induced reduction of BAT thermogenesis through increasing the expression of UCP-1, PGC-1α, PPAR-α and PPAR-γ.

However, it is unclear that whether AuNCs induced changes in BAT thermogenesis were direct effects of AuNCs on BAT or secondary effects of reduced hypothalamic POMC. We have examined whether AuNCs were existed in BAT by using an atomic absorption spectrometer (AAS)^37,57^. As shown in Fig. 4j, AuNCs were present in BAT after intraperitoneal injection of 20 mg/kg AuNCs in rats. Therefore, the pro-thermogenic effect of AuNCs may be a direct effect of AuNCs on BAT. However, due to the influence of the hypothalamus-BAT axis in thermogenesis, we could not exclude the possibility that the effect of AuNCs on BAT thermogenesis may also be a synergic effect of the hypothalamus and BAT.

### AuNCs does not interact with olanzapine directly

As an important principle, the pharmaceutic adjuvant must not affect the efficacy of the major medicines. Usually, the direct interaction between the pharmaceutic adjuvant and the major medicine were used to evaluate whether the pharmaceutic adjuvant would affect the efficacy of the major medicines. In this study, the diphasic titration was employed for quantitative analysis of the interaction between AuNCs and olanzapine by using an UV–Vis spectrometer. In this diphasic titration, AuNCs used as the host subject and olanzapine as the guest object. The original UV–Vis spectra of AuNCs (black curve in Fig. S5a,b) showed obvious absorption peaks at 670 nm, which is the characteristic peak of AuNCs. With the successive addition of olanzapine, the absorption peak intensity of mixture at 670 nm was decreasing slowly (inset in Fig. S5a). However, this slowly decreasing may be attributed to the dilution effect of the solvent. Therefore, we adopted the solvent (DMSO) as the guest object to titrate the AuNCs again. As shown in Fig. S5b, the absorption peak intensity of AuNCs solution were decreasing with the addition of DMSO, and the decreasing degree was almost entirely the same with the diphasic titration of olanzapine. Curve fitting quantification in Fig. S5c showed the difference is within one in ten-thousand. This result indicated that there is no direct interaction between AuNCs and olanzapine.

### AuNCs does not affect the locomotor activity of olanzapine-treated rats

**Figure 5:**
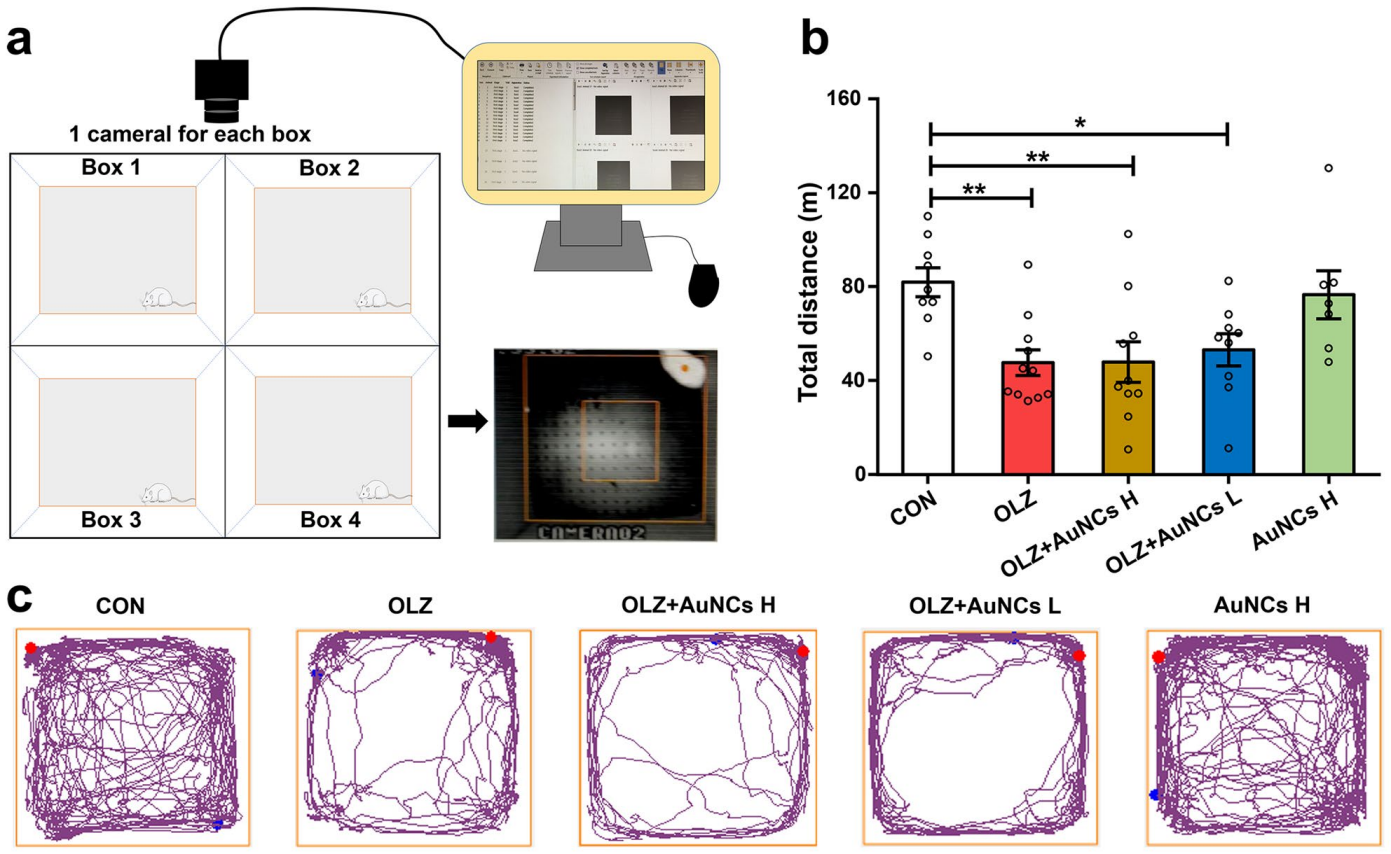
Effects of olanzapine and AuNCs co-treatment on the locomotor activity of rats. (**a**) Experimental procedure of locomotor activity test in rats. (**b**) Total distance of rats in the open field test. (**c**) Examples of locomotor activity rhythm of rats treated with olanzapine and AuNCs. *n* = 7–11/group. All data were presented as mean ± SEM. *p < 0.05, **p < 0.01, OLZ, OLZ + AuNCs H, OLZ + AuNCs L vs*.* CON.

Increased locomotor activity is one of the key markers of schizophrenia^58,59^. In this study, we have examined the effect of AuNCs on rats’ locomotor activity via an open-field test (Fig. 5a). Our results showed that olanzapine reduced the locomotor activity effect in rats, same as previous reports in patients^60^ and rodents^30^. Both high dose and low dose AuNCs co-treatment did not affect the locomotor activity of olanzapine-treated rats (Fig. 5b,c). These results suggested that AuNCs may have a potential to not affect the antipsychotic effect of olanzapine. Further behavioral tests examining aggression and social interaction in rats treated with olanzapine and AuNCs are warranted to understand the effect of AuNCs on olanzapine’s antipsychotic effects.

### AuNCs treatment shows no obvious toxicity

**Figure 6:**
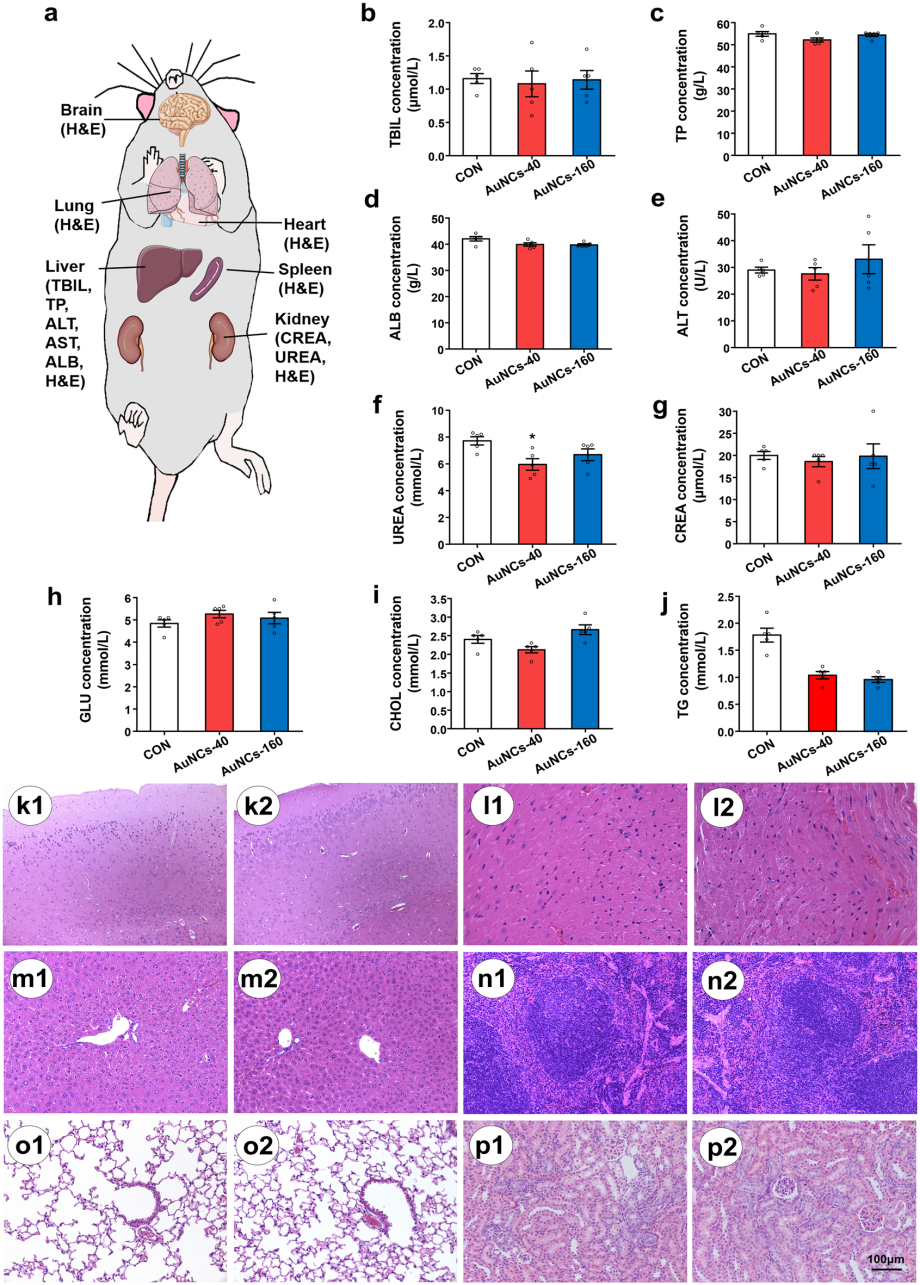
Toxic effects of AuNCs treatment for 28 days in mice. (**a**) Scheme of main organs and plasma biochemical markers that have been examined. (**b**–**j**) Plasma biochemical indexes after 28-day AuNCs treatment at dosages of 40 mg/kg and 160 mg/kg. (**k**–**p**) H&E stanning of mouse tissues including brain (**k**), heart (**l**), liver (**m**), spleen (**n**), lung (**o**) and kidney (**p**) after 28-day treatment of saline (1) or 160 mg/kg AuNCs (2). *n* = 5/group. *TBIL* total bilirubin, *TP* total protein, *ALB* albumin, *ALT* alanine aminotransferase, *CREA* creatinine, *GLU* glucose, *CHOL* cholesterol, *TG* triglycerides.

Our previous work has shown that AuNCs exhibited no toxic effects in vitro in the range of 1–100 mg/L^57^. Here we investigated the toxic effect of AuNCs by short and chronic treatment in mice (Fig. 6). AuNCs administration at 0, 40 and 160 mg/kg for 28 days showed no obvious effects to mouse growth, drinking or locomotor activity. Moreover, it has been found that AuNCs 40 and 160 mg/kg administration for 28 days did not affect the levels of total bilirubin (TBIL), total protein (TP), albumin (ALB), alanine aminotransferase (ALT), urea, creatinine (CREA), glucose (GLU) and cholesterol (CHOL) (Fig. 6b–j). Compared with saline, AuNCs 40 mg/kg slightly reduced the UREA level in the plasma but the UREA level of AuNCs treated mice were within the normal urea range of mice^61^.

H&E staining confirmed that no pathologic lesion or macroscopic changes in the major tissues including brain, heart, liver, spleen, lung and kidney in AuNCs-treated group (Fig. 6k–p). Importantly, to further give evidence for the safety of AuNCs during chronic use, we have examined the chronic toxic effects of AuNCs at a therapeutic dosage (20 mg/kg) administrating for 180 days. We have found that there were no abnormal behaviors in AuNCs treated mice compared with control mice (saline). No obvious pathological changes in the above tissues were observed between AuNCs and saline treated mice (Fig. S6 in SI). These findings illustrated that AuNCs had no obvious toxicity during both short and chronic treatment.

## Discussion

Antipsychotic drug associated weight gain/obesity has become a world-wide issue as the increased prescription of antipsychotics in the clinic. Numerous studies have shown the key role of hypothalamic H1R-AMPK signaling in olanzapine-induced obesity^27^. However, H1 receptor agonists could not pass the blood brain barrier (BBB), which has largely perturbed the use of H1 receptor agonists in the treatment of obesity and related metabolic disorders. The AuNCs synthesized in our group could significantly reverse olanzapine-induced dysfunction of H1R-AMPK signaling in SH-SY5Y cell line, suggesting its potential anti-obesity effect. Therefore, we further examined whether AuNCs could effectively inhibit olanzapine-induced obesity. We have found that AuNCs co-treatment completely prevented and reversed the obesity induced by chronic olanzapine treatment.

**Figure 7:**
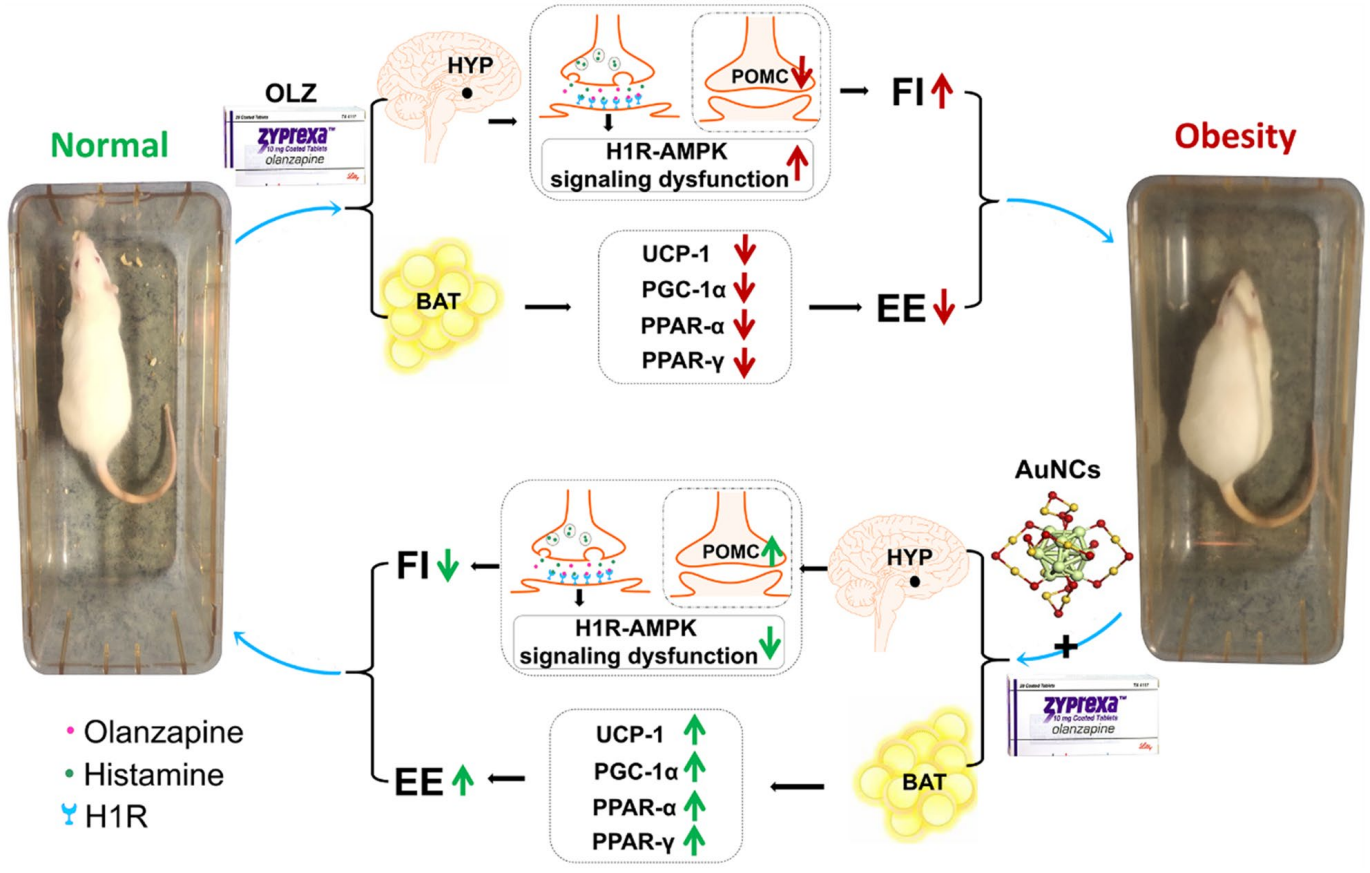
Scheme and potential mechanisms of AuNCs eliminating olanzapine-induced obesity. When olanzapine treated rats were co-treated with AuNCs, AuNCs suppressed hypothalamic H1R-AMPK signaling dysfunction and increasing POMC expression, thus reducing hyperphagia and weight gain. Moreover, AuNCs upregulated the protein expression of UCP-1, PGC-1α, PPAR-α and PPAR-γ in the BAT, and these effects may induce increased energy expenditure and reduced weight gain. *HYP* hypothalamus, *H1R* Histamine H1 receptor, *AMPK* AMP-activated protein kinase, *POMC* proopiomelanocortin, *BAT* brown adipose tissue, *UCP-1* uncoupling protein 1, *PGC-1α* peroxisome proliferator-activated receptor gamma coactivator-1α, *PPAR-α* peroxisome proliferators-activated receptor α, *PPAR-γ* peroxisome proliferators-activated receptor γ, *EE* energy expenditure, *FI* food intake.

Further mechanism investigations revealed that peripheral AuNCs treatment reached the central hypothalamus and effectively ameliorated olanzapine-induced increase in hypothalamic H1R expression to reduce food intake and weight gain. In SH-SY5Y cell line, olanzapine activated AMPK and AuNCs decreased olanzapine-induced increased pAMPK. In rats, olanzapine treatment inhibited AMPK in the hypothalamus. This result was consistent with previous in vivo studies that reported that the hypothalamic AMPK was significantly inhibited during chronic olanzapine treatment^27,47^. AMPK is a well-known energy sensor and negatively responds to positive energy balance. It has been reported that the inhibition of hypothalamic AMPK was due to a feed-back regulation of high energy intake and weight gain during olanzapine treatment^27,47^. In this study, AuNCs treatment reversed olanzapine-induced decrease in pAMPK, suggesting that AuNCs could inhibit olanzapine-induced dysfunction of the hypothalamic H1R-AMPK signaling, therefore reducing weight gain (Fig. 7). Hypothalamic POMC regulate food intake and weight gain via mechanisms that independent with the hypothalamic H1Rs. The immunostaining study showed that olanzapine treatment reduced the hypothalamic Arc POMC expression and these effects were reversed by co-treatment of AuNCs. These findings suggested that POMC-expressing neurons may play an important role in AuNCs’ inhibitory effects on olanzapine-induced obesity. In the hypothalamus, POMC primarily affects energy balance through production of several peptides including alpha-melanocyte stimulating hormone (α-MSH)^62^. However, the effects of olanzapine on hypothalamic α-MSH fibers were not clear. A study in rats demonstrated that olanzapine decreased the α-MSH in the plasma^48^. The α-MSH levels were correlated with POMC mRNA levels in the hypothalamus^48^. These findings suggested that the reduced α-MSH levels in the plasma may due to reduced POMC in the hypothalamus. Therefore, olanzapine-induced obesity mediated by POMC may be related to the production of α-MSH. It has been reported that the anorexigenic effect of α-MSH was associated with the hypothalamic paraventricular nucleus (PVN)^63^. To further understand the role of α-MSH in olanzapine-induced obesity, studies that examine the effect of AuNCs on the PVN α-MSH fibers are needed.

Besides the hypothalamus, AuNCs could also reverse olanzapine-induced reduction in BAT UCP-1 and PGC-1α, which plays an essential role in thermogenesis. The mechanisms are currently unknown. Our study found the presence of AuNCs in BAT, suggesting that AuNCs may directly increase UCP-1 and PGC-1α expression. However, it is known that the hypothalamic H1Rs and POMC could regulate the BAT UCP-1 signaling and therefore mediate BAT thermogenesis^49,64^. Therefore, AuNCs may also modulate the hypothalamic H1Rs and POMC, indirectly leading to increased BAT thermogenesis. It is necessary to further investigate the effect of AuNCs on BAT thermogenesis in H1R knockout (KO) and POMC KO mice. Additionally, PPAR-α and PPAR-γ regulate BAT thermogenesis as transcription factors. Reduced PPAR-α and PPAR-γ could further down-regulate UCP-1 and PGC-1α expression, eventually causing the reduction of percentage of BAT. AuNCs co-treatment dose-dependently increased the expression of PPAR-α and PPAR-γ, indicating that AuNCs could increase BAT thermogenesis at the transcription level (Fig. 7).

Furthermore, in this study, AuNCs evidentially prevented olanzapine-induced weight gain mainly from the 10th day of administration. According to previous studies, olanzapine-induced obesity in rats may show different stages: the early stage (1–2 weeks) with rapid increase of weight gain and hyperphagia, the late stage (> 2 weeks) with slower weight gain without elevated food intake^65,66^. The increase in food intake played a key role in the early stage of olanzapine-induced obesity^27^. In the present study, AuNCs effectively reduced mean food intake mainly appeared in the late period of administration. Therefore, AuNCs did not largely reduce weight gain in the early stage of administration. This is consistent with previous studies reporting that although subjects such as betahistine, metformin and NESS06SM significantly affect the hypothalamic H1Rs, AMPK and POMC, subjects required 5–14 days of treatment to begin to evidentially reduce olanzapine-induced weight gain^28,29,67,68^. Moreover, reductions in BAT temperature and reductions in BAT thermogenesis markers normally occurred in the late stages of olanzapine-induced obesity^30^. AuNCs significantly reduce olanzapine-induced decreased BAT thermogenesis during long-term treatment, resulting in reduced weight gain. Therefore, it is reasonable for AuNCs to start reducing weight gain around day 10 of co-treatment.

In the present study, olanzapine at 3 mg/kg treatment caused slight but significant increases in blood glucose at 30th min (by 18%) and 60th min (by 12%) but not at 90th and 120th min. The fact that there were no changes in blood glucose after 90th and 120th min may have reduced the statistical significance of AUC, resulting in a trend (*p* = 0.1) but no significant rise in AUC when compared to the control group. Consistent with our study, a previous rat study found that olanzapine induced significant increases in blood glucose after 15th and 30th min of glucose administration, but not at 60th and 120th min, with no significant increase in AUC^69^. However, according to the literature, olanzapine treatment at relatively higher doses (8–12 mg/kg) led to higher increases in blood glucose (increased by 30–200%) in rats and mice^70–74^. Therefore, although in the present study there was no significant increase in AUC after 3 mg/kg olanzapine treatment compared with control (*p* = 0.1), the difference between the OLZ and OLZ + AuNCs H groups suggested that AuNCs may have potential effects in reducing glucose metabolism disorder caused by high-dose olanzapine treatment. In further studies, the effect of AuNCs on high dose olanzapine treatment (8–12 mg/kg) induced glucose metabolism disorder and the associated mechanisms will be investigated.

Central H1Rs are also essential for mediating glucose metabolism and inhibiting the development of type II diabetes. In the present study, AuNCs inhibited olanzapine-induced increases in blood glucose. Previous studies have revealed that central histamine H1 receptor activation increased glucose metabolism and reduced high blood glucose via suppression of hepatic signal transducer and activator of transcription-3 (STAT3) and glucose-6-phosphatase (G6Pase)^75^, and this effect is independent of insulin action. Consistent with these findings, AuNCs treatment did not significantly affect insulin levels in the plasma. These results suggested that AuNCs might reduce olanzapine-induced increased blood glucose via the central H1 receptor but not via acting on insulin produce.

The limitation of this study was that we did not examine the effect of AuNCs on BAT temperature due to the equipment limitation. Our previous studies have reported that chronic olanzapine-induced decreased BAT thermogenesis during chronic olanzapine treatment via decreasing UCP1 and PGC-1α expression. Since the importance of AuNCs on the regulation of UCP-1 and PGC-1α, it is suggested that AuNCs could reversed olanzapine-induced decrease in BAT temperature. Moreover, it is worth noting that the toxicity studies were performed in mice rather than rats. The reason is that, according to the literature, mice are commonly used for the toxic effects of gold nanoparticles^76–80^. Moreover, mice were reported to recognize nanoparticles better and generate stronger macrophage responses compared to rats^81^. However, further studies that investigated the toxic effects of AuNCs in rats are warranted.

## Methods

### The synthesis and characterization of AuNCs

A typical NIBC molecule modified AuNCs with a diameter lower than 2 nm was synthesized in one-pot by using a two-step method according to our previous works^37,38,41^. Firstly, the *N*-isobutyryl-*L*-cysteine (NIBC) and HAuC_l4_ were added into a cooled methanol solution at 0 °C (cool bath). Then the mixtures were incubated in cool bath for 1 h with a gently stirring to get a react intermediates of Au^+^(NIBC) complex. Secondly, NaBH_4_ was added into the mixtures and incubated for another 0.5 h with a strongly stirring in room temperature to get the final products of AuNCs. After dialysis and lyophilization, the pure AuNCs were characterized by the TEM, UV–Vis–NIR absorption spectroscopy, XPS and FT-IR spectroscopy. The *N*-isobutyryl-*L*-cysteine was chosen as the ligand of AuNCs based on the following reasons. On one hand, our previous studies have shown that gold nanoclusters modified with *N*-isobutyryl-*L*-cysteine (NIBC-AuNCs) has shown good biocompatibility in both cell and mice, and could penetrate BBB. On the other hand, N-terminal aminated cysteine such as *N*-acetyl-*L*-cysteine could increase histamine secretion and suppress the activation of AMPK as well as inhibit glucose intolerance^82–85^. However, how *N*-isobutyryl-*L*-cysteine affect the AMPK signal signaling pathway remains to be further explored. Therefore, *N*-isobutyryl-*L*-cysteine were chosen for the ligand of AuNCs.

### Investigation of the interaction between olanzapine and AuNCs via UV–Vis titration

UV–Vis titration experiments were adopted to investigate the interaction between olanzapine molecules and AuNCs. UV–Vis titration measurements were carried out in a Shimadzu UV-1800 UV–Vis spectrometer with a 1 cm path-length quartz cuvette. The host samples solutions of AuNCs in dimethyl sulfoxide (DMSO) with a concentration of 500 μg/mL were prepared at room temperature (about 25 °C). The guest solution of 10,000 μg/mL olanzapine in DMSO were also prepared at room temperature. The mother liquor of 3.0 mL of AuNCs solution were titrated by successive additions of fixed small aliquots (10 μL) of the guest olanzapine solution. Titrations were done manually by using trace syringes, and the UV–Vis intensity was measured after every drops. The UV–Vis adsorption peak of AuNCs in DMSO was also at 670 nm, same to that in water. 36 times of titrations were carried out in this study. In order to exclude the decrease of UV–Vis peak intensity caused by solvent dilution, a blank control group was adopted by using DMSO solvent as the guest solution. The mother liquor of 3.0 mL of AuNCs solution were titrated by successive additions of fixed small aliquots (10 μL) of DMSO.

### Cell line and olanzapine powder

SH-SY5Y cell line was purchased from Bluef (Shanghai) Biotechnology Development Co., Ltd, Shanghai, China. The SH-SY5Y were cultured in Dulbecco's Modified Eagle Medium /Nutrient Mixture F-12 (DMEM/F12) (BL305A, Lanjike Technology Co., Ltd, Anhui, China), 15–20% fetal bovine serum (11011-8611, Zhejiang Tianhang Biological Technology Co., Ltd, Zhejiang, China) and 1% penicillin/streptomycin (A3160801 and 15140-122, Thermo Fisher Scientific (China) Co., Ltd, Shanghai, China), at 37 °C in a humidified 5% CO_2_ incubator. Olanzapine powder was purchased from Sigma (LRAC0272) and was dissolved in Dimethyl sulfoxide (DMSO). Control cells were treated with DMEM + DMSO (vehicle). Cells were treated for 2 h and 24 h.

### The effect of AuNCs on olanzapine-induced H1R-AMPK signal dysfunction in vitro

The effect of olanzapine and AuNCs on H1R and pAMPK signaling in cells were examined by immunostaining. In brief, SH-SY5Y cells were plated in 6-well plates on a glass coverslip coated with poly-d-lysine, grown at the corresponding temperatures (37 °C in a humidified 5% CO_2_). Cells were divided into five groups (*n* = 4/group) and treated with vehicle, olanzapine-50 μM, olanzapine-50 μM + AuNCs-20 mg/L, olanzapine-50 μM + AuNCs-10 mg/L, AuNCs-20 mg/L for 2 h and 24 h. Cells were fixed with 4% paraformaldehyde (PFA), washed with PBS, repaired with EDTA antigen retrieval buffer (Beijing Solarbio Science & Technology, C1034) and blocked with 10% BSA. Then, cells were incubated with primary antibodies (H1R and pAMPK (bs-6663R, bsm-52132R, Beijing Biosynthesis Biotechnology). The cells were incubated with goat anti-rabbit secondary antibodies (ab6717, Abcam Trading; GB21303, Wuhan Google Biotechnology). The nucleus was counterstained by DAPI (C0065, Beijing Solarbio Technology) for 10 min at room temperature in the dark. The percentage area of the H1R and pAMPK over the total area was measured by the software Image-pro plus 6.0 (Media Cybernetics, Inc., Rockville, MD, USA).

## Animals and olanzapine tablets

Female Sprague Dawley (SD) rats (9–10 weeks) were obtained from the Animal resources center (SPF (Beijing) Biotechnology Co., Ltd, Beijing, China) and Hubei experimental animal research center (Hubei, China). Female C57BL/6 and BALB/c mice (weight 18–20 g) were purchased from Hubei experimental animal research center and Zhejiang weitong lihua experimental animal technology Co. Ltd., China. Animals were housed in barrier system (22 ± 2 °C on a 12 h light–dark cycle, lights on 07:00). All the animals were fed a standard diet throughout the studies. Olanzapine tablets was purchased from Eli Lilly, S.A. Madrid, Spain. The animal experiments were approved by the Animal Ethics Committee of Wuhan University of Technology (SYXK 2017-0092), and carried out according to the institutional guidelines. All experiments performed were following relevant guidelines and regulations. In addition, all experiments were carried out in compliance with the ARRIVE guidelines.

### The preventive effect of AuNCs on olanzapine-induced weight gain in rats

Rats were divided into 5 groups randomly (*n* = 10–12/group): control (CON, group1), olanzapine-only (OLZ, group 2), co-treatment of olanzapine and AuNCs high dose (OLZ + AuNCs H, group 3), co-treatment of olanzapine and AuNCs low dose (OLZ + AuNCs L, group 4), AuNCs high dose-only (AuNCs H, group 5). Olanzapine was mixed with 0.3 g cookie-dough pellet according to our previous protocol^30^. From the beginning of the experiment, rats in group 2–4 received oral olanzapine administration (1 mg/kg, t.i.d, at 07:00, 15:00 and 23:00). Rats in group 1 and group 5 were given vehicle (cookie with no olanzapine) as control. From the first day of olanzapine (or vehicle) administration, rats in OLZ + AuNCs H and OLZ + AuNCs L groups received 20 mg/kg or 10 mg/kg AuNCs treatment through IP injection (once a day). Rats in CON and OLZ group received an IP injection of saline. Rats in AuNCs high dose-only group were treated with 20 mg/kg AuNCs as a drug control group. The above treatment was last for 21 days. Food intake was measured every 24 h and weight gain was measured every 48 h. After the last treatment, rats were euthanized by fast CO_2_ infusion^32^. The plasma was collected and stored in – 80 °C. BAT and hypothalamus were dissected stored in − 80 °C. Half of the BAT tissues were fixed in 4% PFA and used for immunohistochemistry analysis.

### The therapeutical effect of AuNCs on established obesity induced by olanzapine

Rats were divided into two groups including olanzapine (OLZ, *n* = 33) and control (CON, *n* = 22) groups. The rats in CON group were treated with vehicle, and rats in OLZ group were treated with olanzapine the same as in Animal experiment 1 for 28 days. From day 29, olanzapine treated rats were randomly divided in to three groups including olanzapine only (OLZ), olanzapine + AuNCs high dose (OLZ + AuNCs H, 20 mg/kg), olanzapine + AuNCs low dose (OLZ + AuNCs L, 10 mg/kg) groups and were treated with AuNCs and olanzapine based on grouping for 28 days. The rats treated with vehicle were randomly divided into 2 groups including control (CON) group and AuNCs high dose (AuNCs H, 20 mg/kg, IP) group. Rats in CON group were IP injected with saline and rats in AuNCs H were IP injected with 20 mg/kg AuNCs for 28 days. The body weight and food intake of each rat was measured every 48 h and 24 h, respectively.

### Investigation of the toxicity of AuNCs during short-term and long-term treatment

In short-term treatment, 15 female mice were randomly divided into 3 groups: control, AuNCs 40 mg/kg and AuNCs 160 mg/kg (*n* = 5/group). The mice received AuNCs or saline treatment by IP injection for 28 days (once a day). Mouse behaviors were observed every day. After the last treatment, mice were sacrificed and the blood were collected. The biochemistry markers including TBIL, TP, ALB, ALT, UREA, CREA, GLU and CHOL were examined by using a biochemical analyzer. In long-term treatment, 15 female mice were randomly divided into control group and AuNCs group. The mice received a therapeutical dose of AuNCs (20 mg/kg) or saline treatment by IP injection (once a day) for 6 months. After the last treatment, mice were sacrificed and tissues including brain, heart, liver, spleen, lung, and kidney were collected and used for histology.

### Glucose intolerance test

Rats were fasted for 16 h with free of water. Blood was collected from the tail vein of rats, and a fasting blood glucose (0 min) was measured using a blood glucose meter (Johnson & Johnson one touch ultra, Johnson & Johnson (China) Medical Equipment Co., Ltd.). Then rats were IP injected with 1 g/kg glucose. The blood glucose at 30th min, 60th min, 90th min and 120th min post injection was detected. The area under curve (AUC) of glucose level was calculated for each rat.

### Plasma hormone analysis and biochemical analysis

Levels of leptin, insulin, cholesterol and triglycerides in the plasma were analyzed by commercially available ELISA Kits (leptin, Elabscience Biotechnology Co., Ltd., Hubei, China, E-EL-R0582c; insulin, E-EL-R2466c, cholesterol, Changchun Huili Biotechnology Co., Ltd., Jilin, China, C048-a; triglycerides, C019-a). The absorbance of the samples was measured with a FlexStation 3 Multi-Mode Microplate Reader (Molecular Devices).

### Open filed test

Rats were subjected to open field behavioral testing to examine the locomotor activity of each rat. In brief, rats were put into an open field experiment box (60 cm × 60 cm × 40 cm). A high-frequency camera was used to track and record the behavior changes of rats within 30 min on free movement in the open field equipment. Total distance travelled (m) were recorded. Feces of each rat was cleaned after the experiment, and the bottom surface was cleaned with 75% ethanol solution and wiped to prevent odor infection.

### Histology (H&E staining)

To access the morphology of the BAT adipocytes in olanzapine and AuNCs treated rats, the PFA-embedded BAT tissue was section-sliced (4 μm/section) and mounted on glass slides (*n* = 4/group). Two fields per section were randomly captured with a Nikon camera (Eclipse ci, Nikon Instruments (Shanghai) Co., Ltd., Shanghai, China) at 10× objective. The percentage area of the multilocular brown adipocytes over the total area was measured by the software Image-pro plus 6.0 (Media Cybernetics, Inc., Rockville, MD, USA). To investigate the toxicity of AuNCs on mice, PFA- embedded tissues were sectioned by Leica pathology microtome (4 μm/section) and treated with hematoxylin and eosin-staining immediately (*n* = 3/group). The histological sections were examined by biological microscope (Olympus) with 20× objective lens.

### Western blot procedures

The protein expression of key molecules of the H1R-AMPK signaling, POMC and UCP-1 signaling were examined by western blot. In brief, tissues were homogenized and the protein concentration was determined by BCA protein concentration assay. Protein was separated and then transferred onto polyvinylidene difluoride membranes and incubated with primary antibodies (H1R, Wuhan Sanying Biotechnology, 13413-1-ap; AMPK, Affinity, Df6361; pAMPK, Affinity, Af3423; POMC, Wuhan Sanying Biotechnology, 66358-1-ig; UCP-1, Wuhan Sanying Biotechnology, 23673-1-ap; PGC-1-α, Wuhan Sanying Biotechnology, 20658-1-ap; PPAR-α, Wuhan Sanying Biotechnology, 15540-1-ap; PPAR-γ, Wuhan Sanying Biotechnology, 16643-1-AP) and secondary antibodies (BOSTER Biological Technology, BA1051 and BA1054). The enhanced chemiluminescence (ECL) kit were used to analyze the band. The quantification of protein was normalized to those of β-actin (BOSTER Biological Technology, BM0627).

### Detection of AuNCs content in BAT

Six female (*n* = 3/group) rats received intraperitoneal injection of 20 mg/kg AuNCs or saline. After 2 h of administration, rats were sacrificed and BAT were quickly collected and weighed. The BAT was homogenized and digested by nitric acid and aqua regia. The AuNCs content were detect by atomic absorption spectrometer (Analytik Jena, Germany, ContrAA800) according to the procedure of our previous studies^37,57^.

### Immunofluorescence staining of POMC in rat hypothalamus

The rat brain was extracted and fixed in 4% PFA. After dehydrating, clearing, and dipping in paraffin, tissues were embedded in paraffin and sectioned (4 µm) using Leica pathology microtome. Sections were blocked with 10% BSA at room temperature for 30 min. The sections were then incubated with primary antibody (66358-1-Ig, Wuhan Sanying Biotechnology Co., Ltd.,) and then washed with PBS and incubated with the secondary antibodies (BA1031, Wuhan Boster Biological Engineering Co., Ltd.,). The immunofluorescence labeling of POMC in the hypothalamus was observed using fluorescent microscope (Olympus). The POMC fluorescence intensity was quantified by Image J software.

### Immunohistochemistry

Immunohistochemistry was used to examined the expression of UCP-1and PGC-1 in BAT according to the previous work^37^. BAT sections were incubated with 3% hydrogen peroxide solution in the dark at room temperature, and were blocked with 3% BSA. The sections were then incubated with the primary antibodies and secondary antibodies (UCP-1, Wuhan servicebio technology, GB11370-1; PGC-1α, Wuhan servicebio technology, GB14097). The sections were then counterstained with haematoxylin. The UCP-1 and PGC-1α were counted by ratio of positive cells to total tissue area in each field by the software Image-pro plus 6.0 (Media Cybernetics, Inc., Rockville, MD, USA).

### Statistical analysis

SPSS 22.0 (IBM SPSS Statistics, USA) was used for statistical analysis. The distributions of data were detected by Kolmogorov–Smirnov test. The statistical differences of normal distributed data were analyzed by one-way ANOVA with followed by post hoc Dunnett t test for multiple comparisons. Pearson’s correlation test was used to analyze the relationships among the measurements. The data was expressed as Mean ± SEM. *p* < 0.05 was considered statistically significant.

## Supplementary Information

Supplementary Information.

## Data Availability

All relevant data supporting the key findings of this study are available within the article and its Supplementary Information files or from the corresponding author upon reasonable request.
