# Supplementary material for: Gold nanoclusters eliminate obesity induced by antipsychotics
Source: Sci Rep. 2022 Apr 1;12:5502. doi: 10.1038/s41598-022-09541-x (PMC8975852; doi:10.1038/s41598-022-09541-x)
Supplement: Supplementary file 1 — Supplementary Information. [file 41598_2022_9541_MOESM1_ESM.docx]

Supporting Information

**Gold Nanoclusters Eliminate Obesity Induced by Antipsychotics**

**Meng He^1,+^, Jing Yao^1,+^, Zijun Zhang^2,+^, Ying Zhang^1^, Rui Chen^2^, Zhenhua Gu^2^, XuFeng Huang^3^, Chao Deng^3^, Ruqin Zhou^1^, Jun Fan^1^, Baohua Zhang^4^, Yanqian Xie^1^, Guanbin Gao^2*^ and Taolei Sun^1,2*^**

^1^School of Chemistry, Chemical Engineering and Life Sciences, Wuhan University of Technology, Wuhan, 430070, China.

^2^State Key Laboratory of Advanced Technology for Materials Synthesis and Processing, Wuhan University of Technology, Wuhan, 430070, China.

^3^School of Medicine and Molecular Horizons, University of Wollongong, NSW, 2522, Australia.

^4^The National Clinical Research Center for Mental Disorders & Beijing Key Laboratory of Mental Disorders, Capital Medical University, Beijing, 100191, China.

*Corresponding authors:

Professor Guanbin Gao, PhD: [gbgao@whut.edu.cn](mailto:gbgao@whut.edu.cn);

Professor Taolei Sun, PhD: [suntl@whut.edu.cn](mailto:suntl@whut.edu.cn);

^+^These authors contribute equally to this work.

**Contents**

**1. Supplementary figures.**

Fig S1. Effects of olanzapine and AuNCs co-treatment on H1R-AMPK signaling in cultured SH-SY5Y cells.

Fig S2. Effects of olanzapine and AuNCs co-treatment on white adipose tissue.

Fig S3. Effects of olanzapine and AuNCs co-treatment on plasma triglycerides and cholesterol levels.

Fig S4. Effects of olanzapine and AuNCs co-treatment on leptin and insulin levels.

Fig S5**.** AuNCs had no interaction with olanzapine *in vitro*.

Fig S6. Toxic effects of AuNCs treatment for 180 days in mice.

**2. Fig S7. Original figures of rats in Fig 2d.**

**3. Fig S8. Original figures in Fig 3b.**

**4. Fig S9. Original figures in Fig 4a and 4f.**

**5. Supplemental Statistical Section****.**

1. Supplementary figures

**
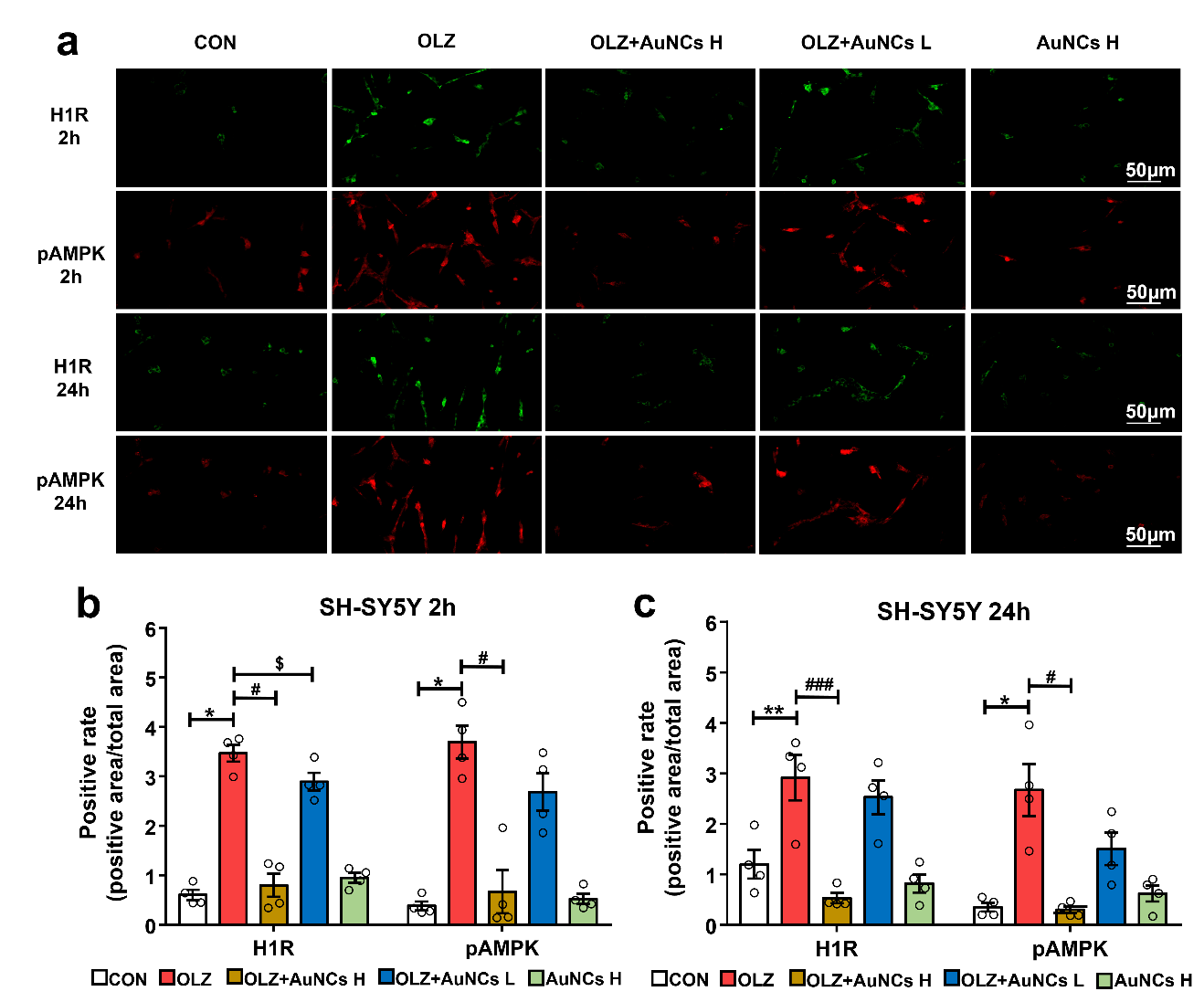
**

Fig S1. Effects of olanzapine and AuNCs co-treatment on H1R-AMPK signaling in cultured SH-SY5Y cells. (a) Effects of 2 h and 24 h olanzapine (50 μM) and AuNCs (high dose: 20 mg/L; low dose: 10 mg/L) co-treatment on H1R and pAMPK immunoﬂuorescence density in cultured SH-SY5Y cells. (b and c) The corresponding quantiﬁcation of relative ﬂuorescence intensity for H1R and pAMPK. *n* = 4/group. All data were presented as mean ± SEM. **p*< 0.05*,* ***p*< 0.01, OLZ *vs.* CON; ^#^*p* < 0.05, ^###^*p* < 0.0001, OLZ + AuNCs H *vs.* OLZ; ^$^*p*< 0.05, OLZ + AuNCs L *vs.* OLZ.


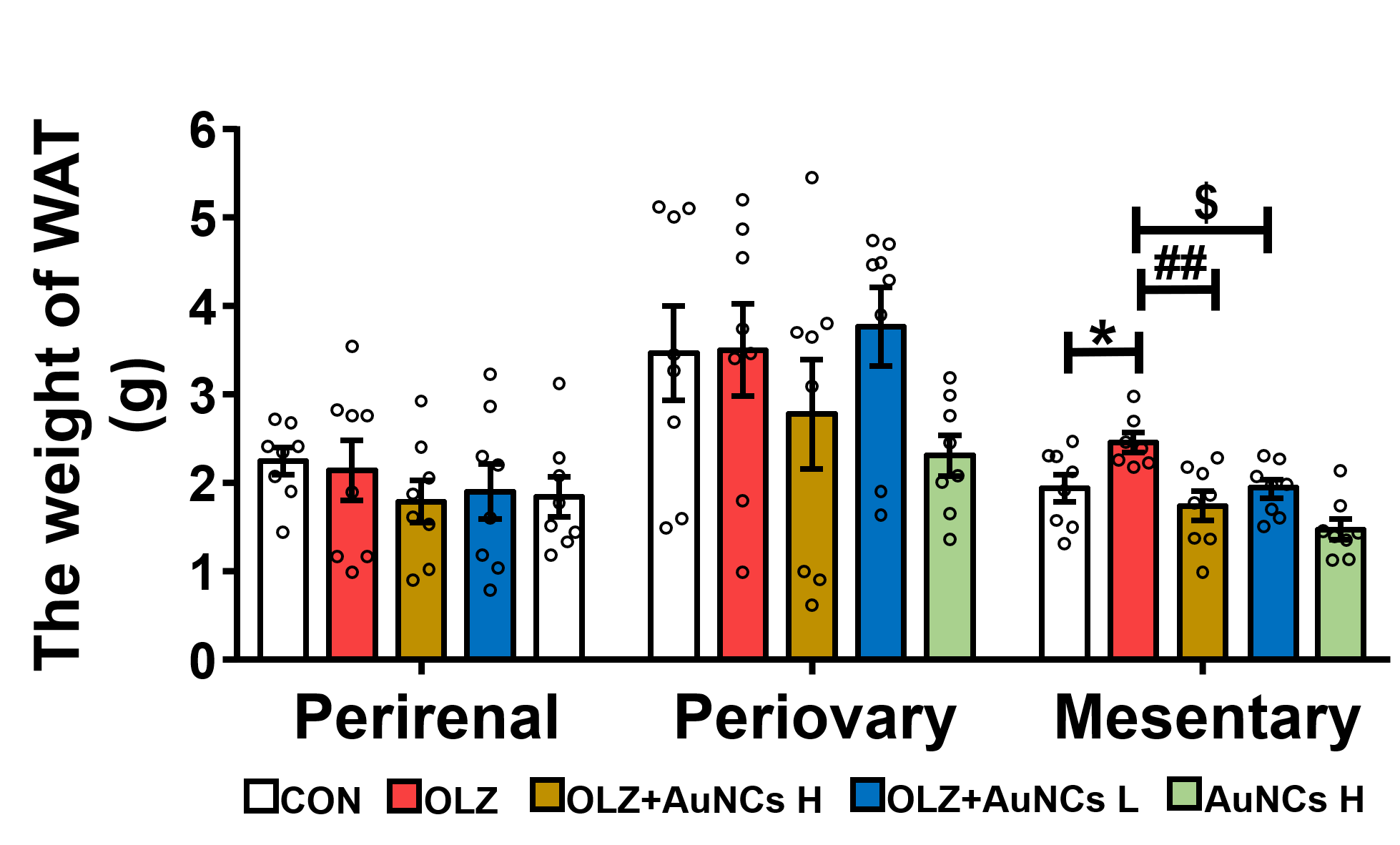


Fig S2. Effects of olanzapine and AuNCs co-treatment on white adipose tissue. *n* = 8/group. All data were presented as mean ± SEM. **p*< 0.05, OLZ *vs*. CON; ^##^*p* < 0.01, OLZ + AuNCs H *vs.* OLZ; ^$^*p* < 0.05, OLZ + AuNCs L *vs.* OLZ


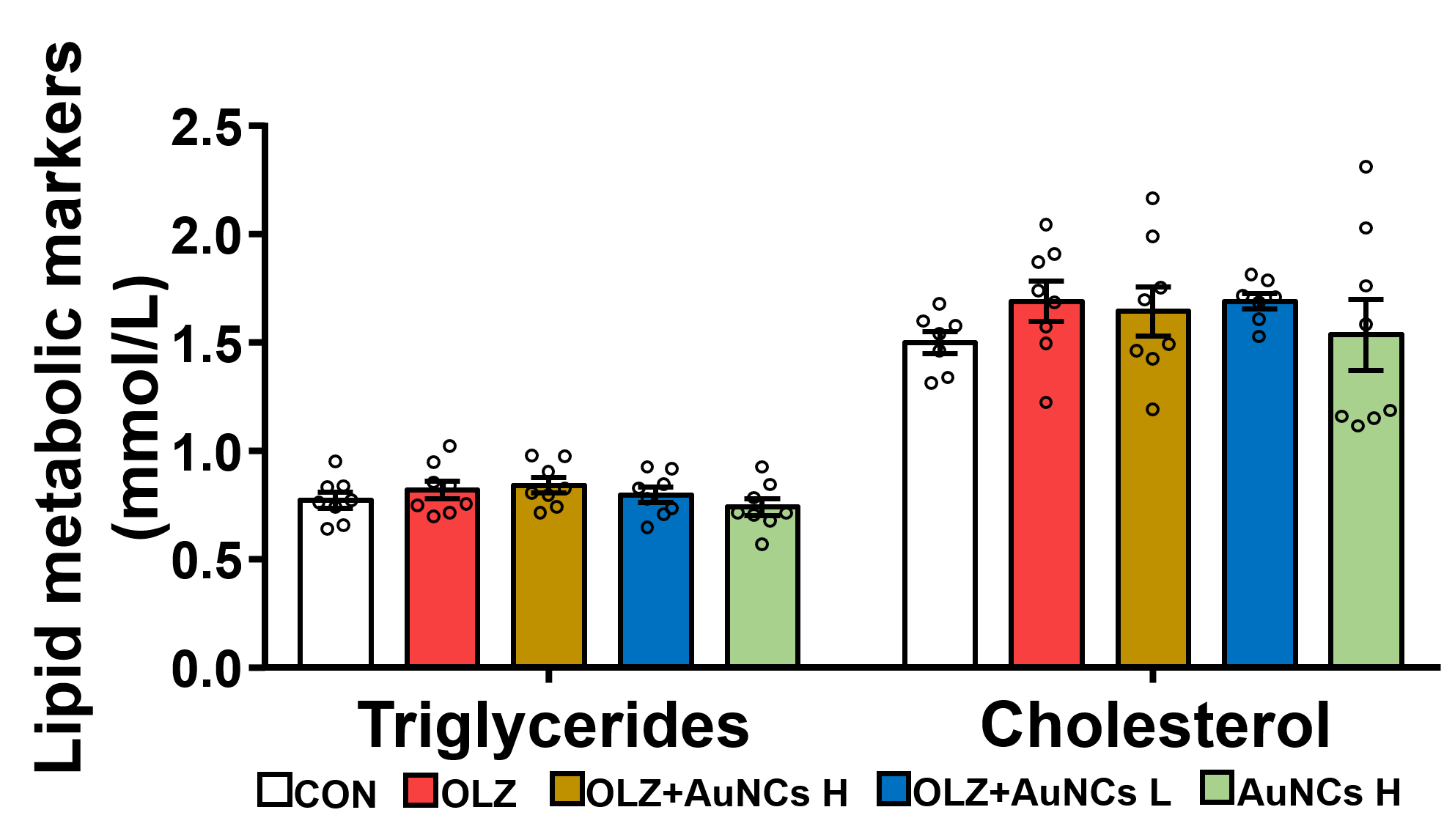


**Fig S3.** Effects of olanzapine and AuNCs co-treatment on plasma triglycerides and cholesterol levels. *n* = 8/group. All data were presented as mean ± SEM.


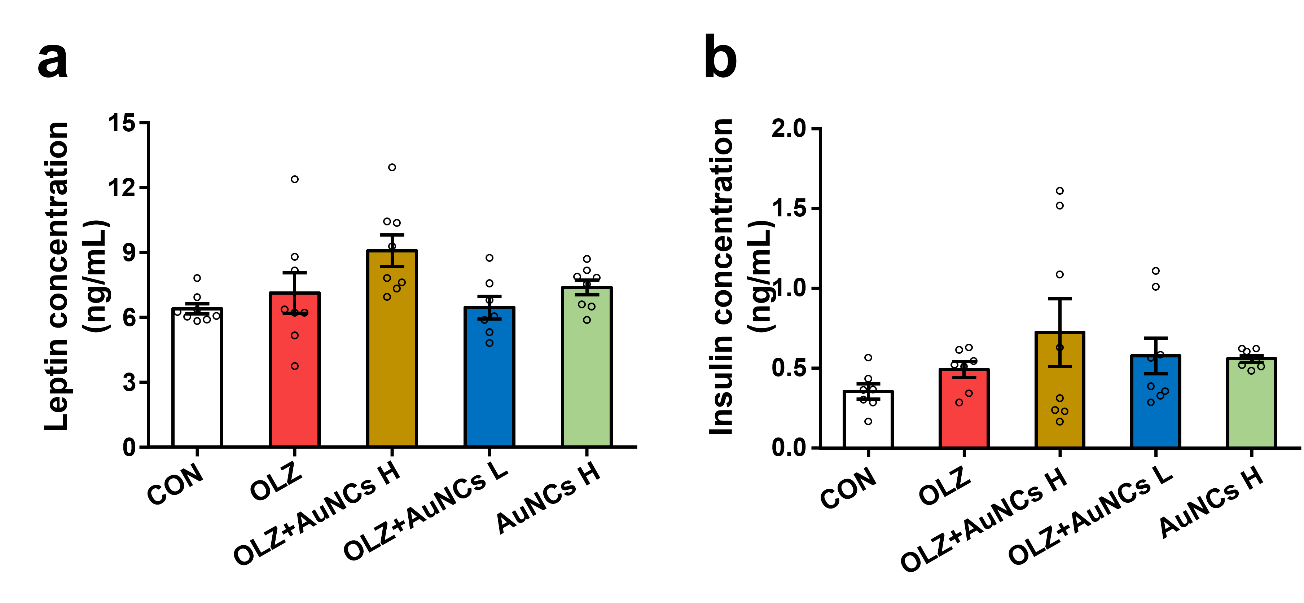


**Fig S4.** Effects of olanzapine and AuNCs co-treatment on leptin and insulin levels. *n* = 8/group. All data were presented as mean ± SEM.


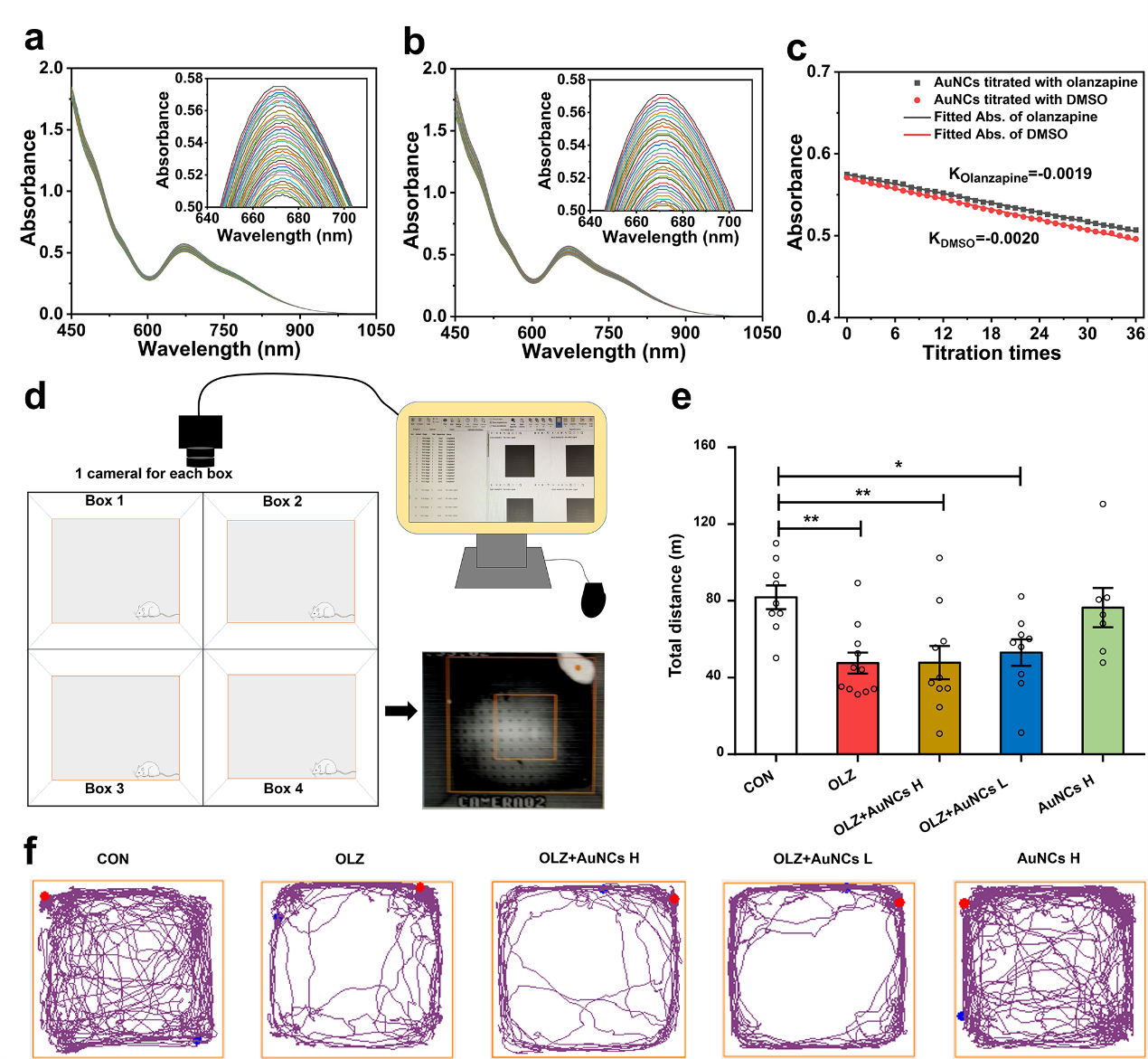


**Fig S5.** AuNCs had no interaction with olanzapine *in vitro*. (a-b) The UV-vis absorption spectra vs time of AuNCs titrated with olanzapine (a) or DMSO (b), the inset of E1 and E2 are the corresponding magnifications. (c) The UV-vis spectra intensity at 670 nm vs titration times of AuNCs titrated with olanzapine (black line) or DMSO (red line).

**
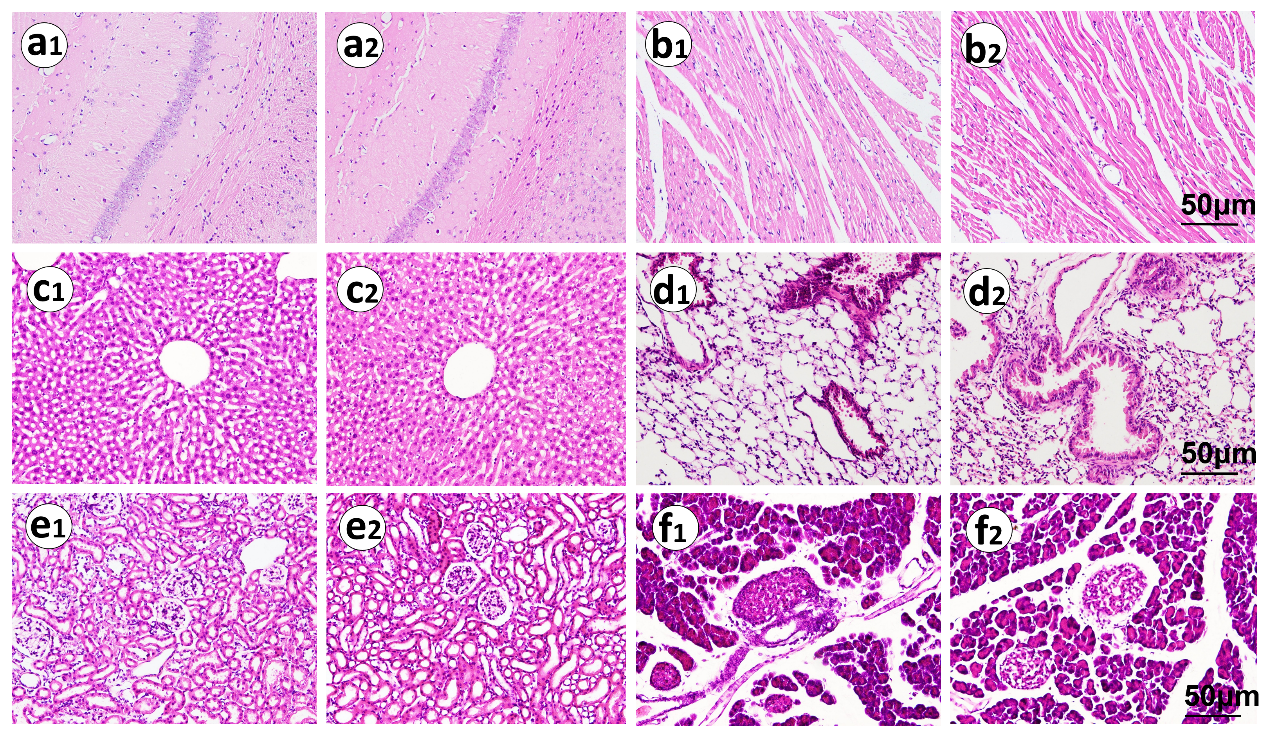
**

**Fig S6.** Toxic effects of AuNCs treatment for 180 days in mice. The H&E stanning of mouse tissues including brain (a), heart (b), liver (c), lung (d), kidney (e) and pancreas (f) after 180-day of saline (1) or 20 mg/kg AuNCs (2) treatment. *n* = 3/group.

**2.Fig S7. Original figures of rats in Fig 2d.** Effects of AuNCs on established obesity induced by olanzapine treatment in rats.

**Day0:**

**
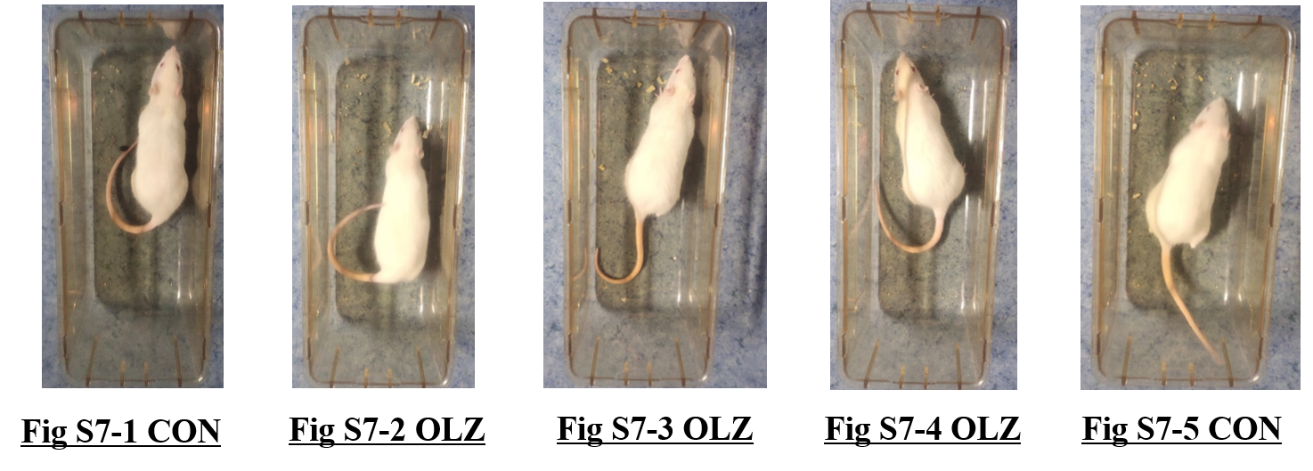
**

**Day28:
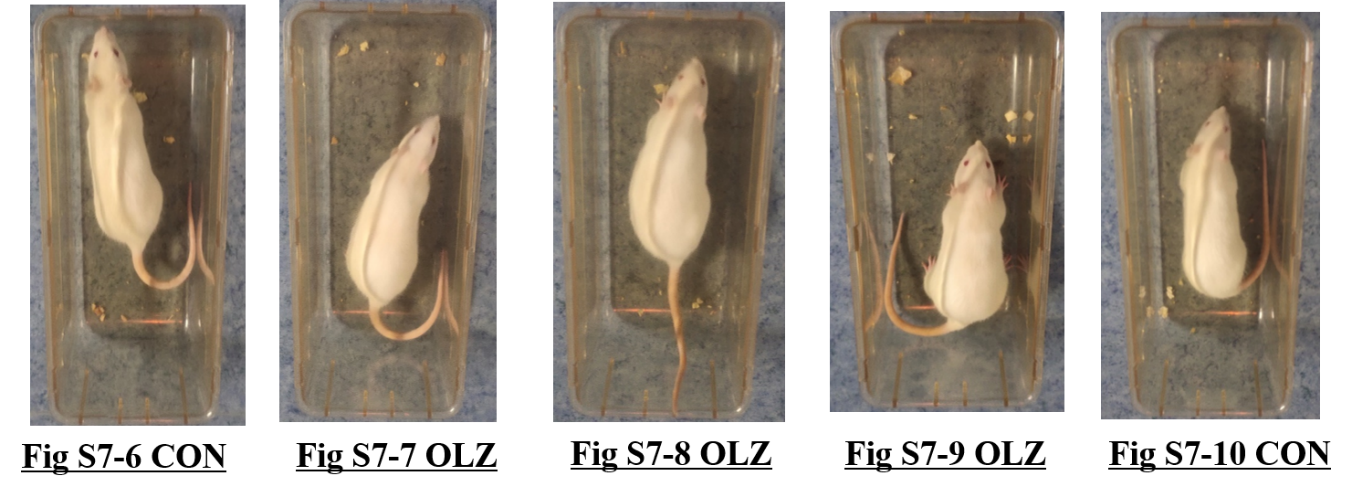
**

**Day49:**

**
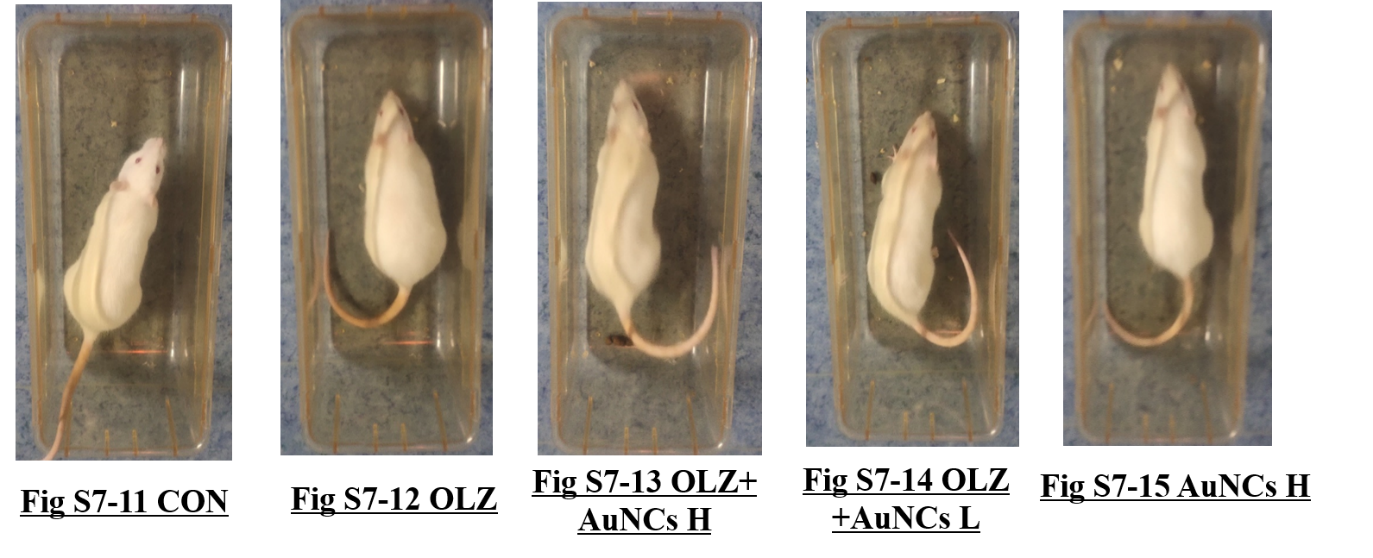
**

**3. Fig S8. Original figures in Fig 3b** Representative western blot figures of H1R, AMPK, pAMPK and POMC in the hypothalamus after co-treatment of olanzapine and AuNCs.

**AMPK**

**pAMPK**

**β-actin**

**H1R**

**POMC**

**CON**

**OLZ**

**OLZ+**

**AuNCs H**

**OLZ+**

**AuNCs L**

**AuNCs H**


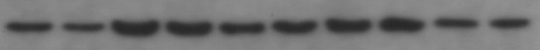

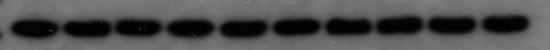

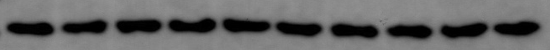

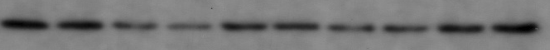

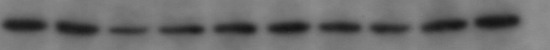


**Fig S8-1 H1R：**


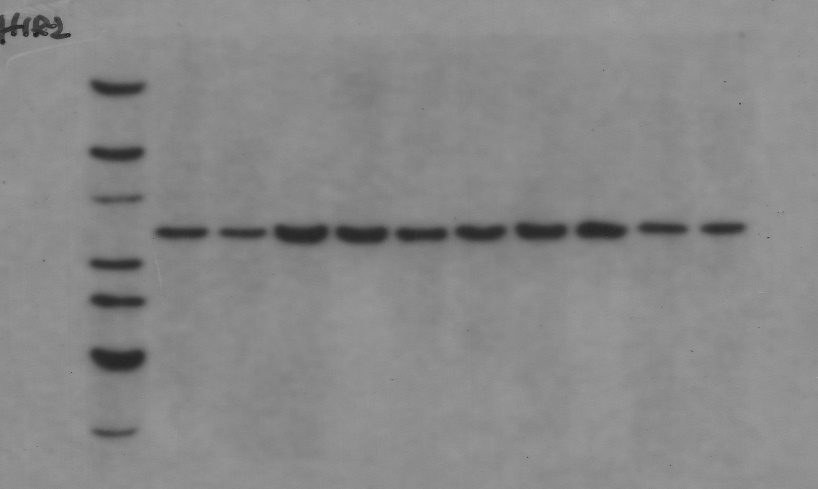


60kDa

50kDa

40kDa

30kDa

20kDa

**56kDa**

120kDa

80kDa

**Fig S8-2 AMPK：**


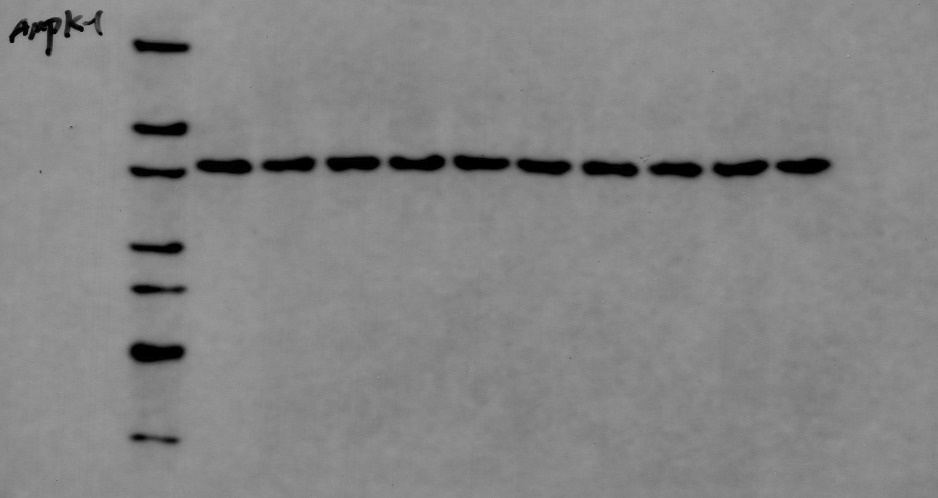


**63kDa**

**Fig S8-3 pAMPK**

**62kDa**


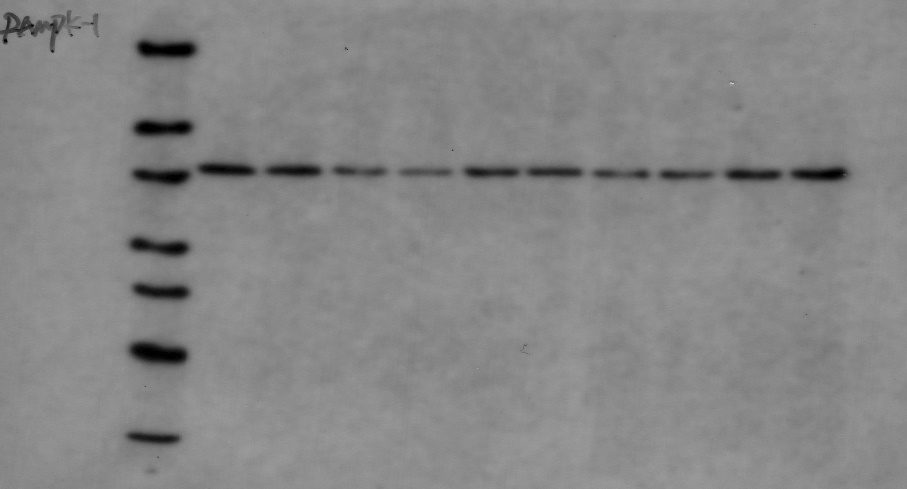


**Fig S8-4 POMC：**

**35kDa**


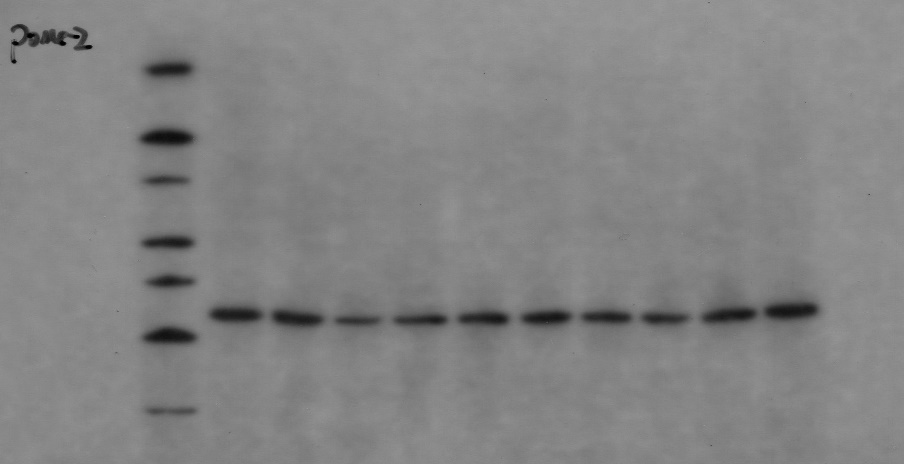


**Fig S8-5 β-actin：**

**42kDa**


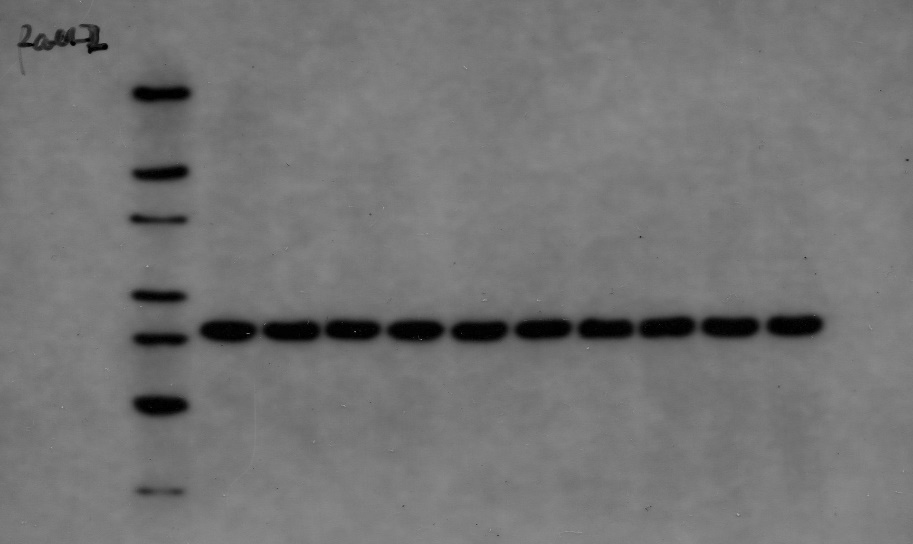


**4.Fig S9. Original figures in Fig 4a and 4f.**

**Fig 4a** Representative western blot figures of UCP-1, PGC-1α, PPAR-α, PPAR-γ expression in BAT.

**PGC-1α**

**AuNCs H**

**OLZ+**

**AuNCs L**

**PPAR-γ**

**β-actin**

**UCP-1**

**CON**

**OLZ**

**OLZ+**

**AuNCs H**

**A**


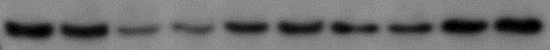

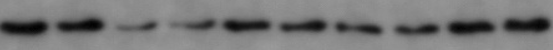

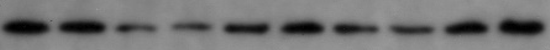

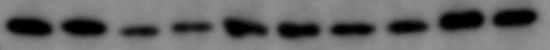

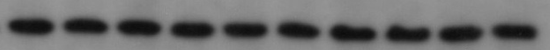


**PPAR-α**

**Fig S9-1 UCP-1:**

**33kDa**


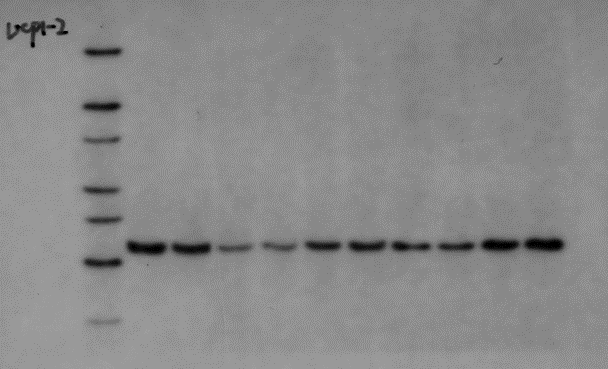


**Fig S9-2 PGC-1α：**

**91kDa**


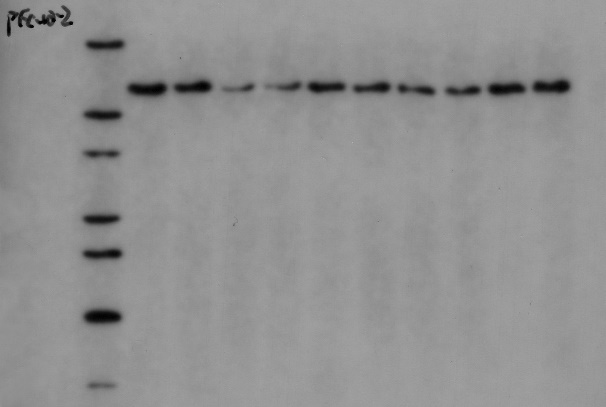


**Fig S9-3 PPAR-α：**

**52kDa**


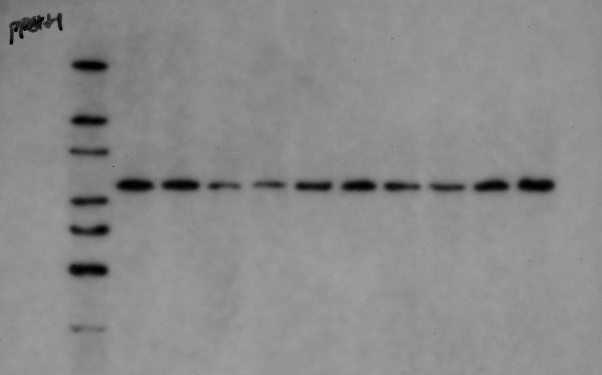


**Fig S9-4 PPAR-γ：**

**57kDa**


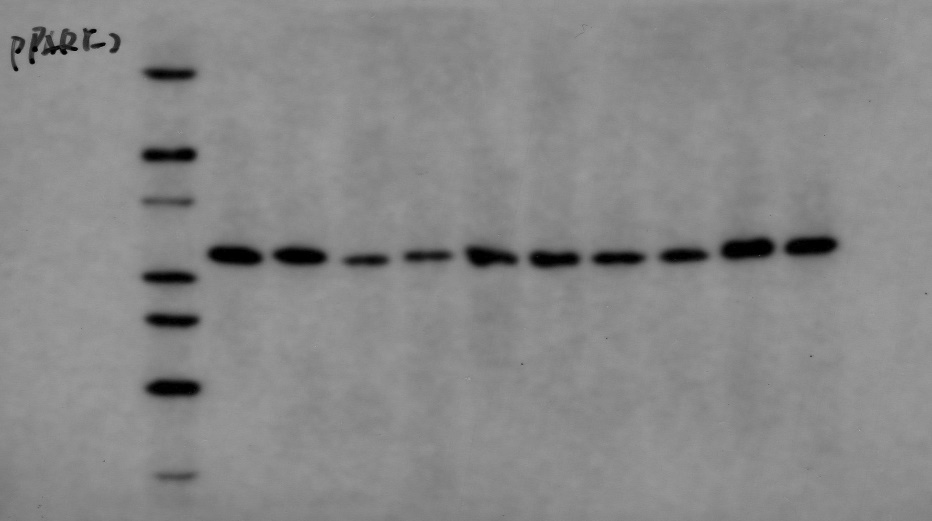


**Fig S9-5 β-actin：**

**42kDa**


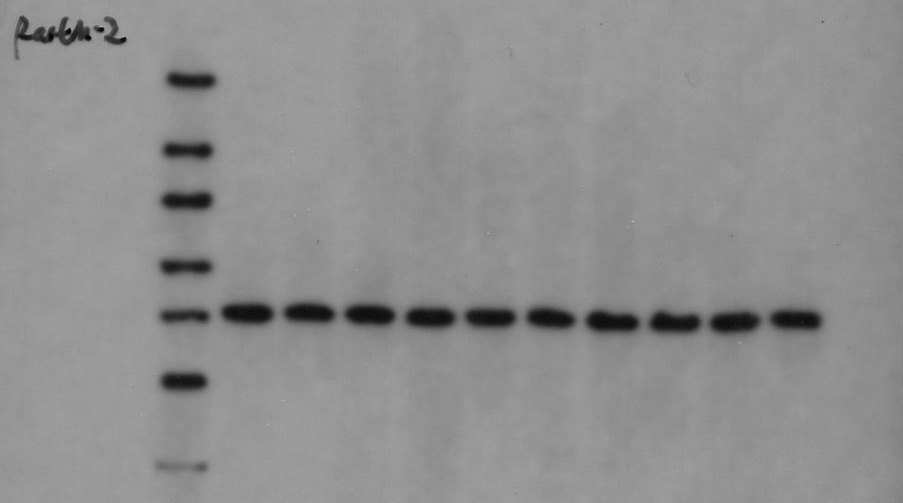


**Fig 4f. Immunohistochemistry of UCP-1**

**Fig S9-6 CON**


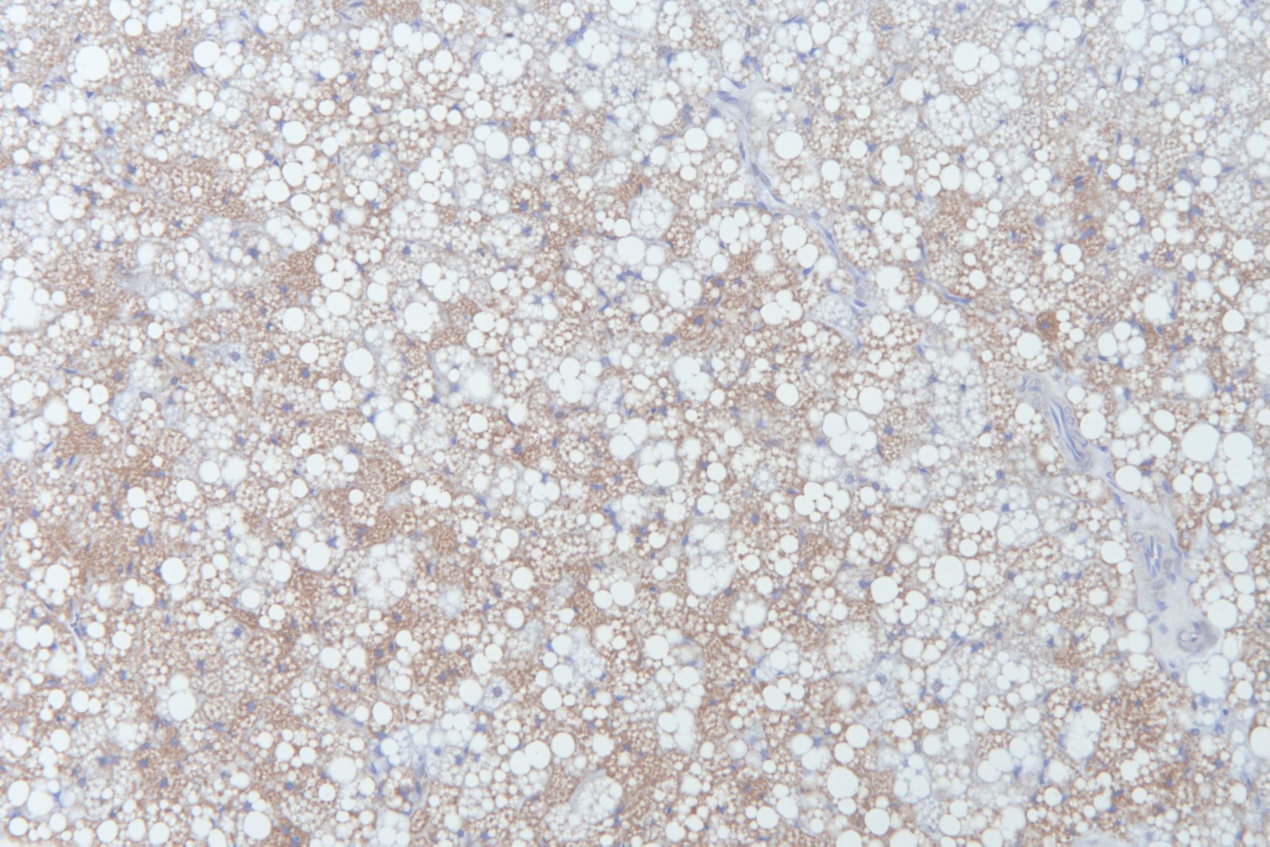


**Fig S9-7 OLZ**


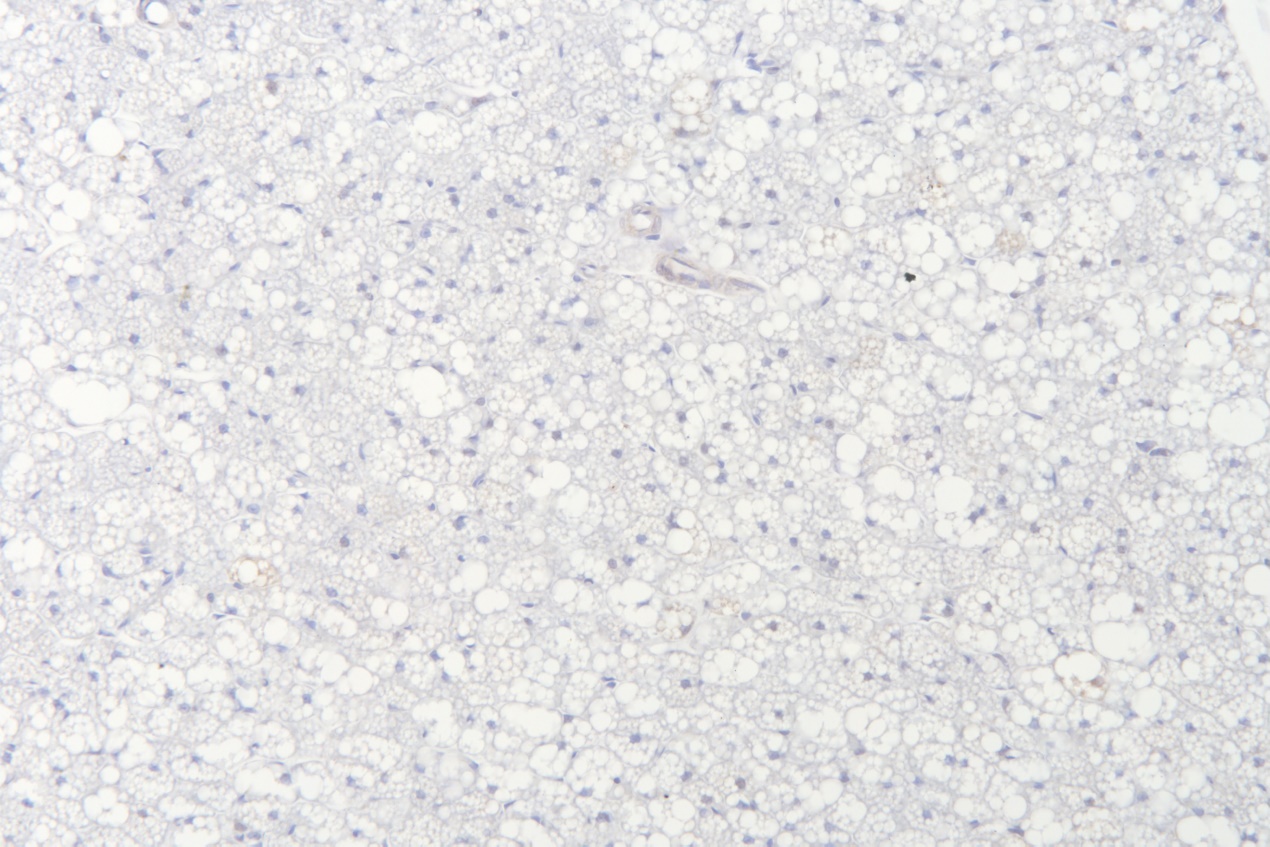


**Fig S9-8 OLZ+ AuNCs H**


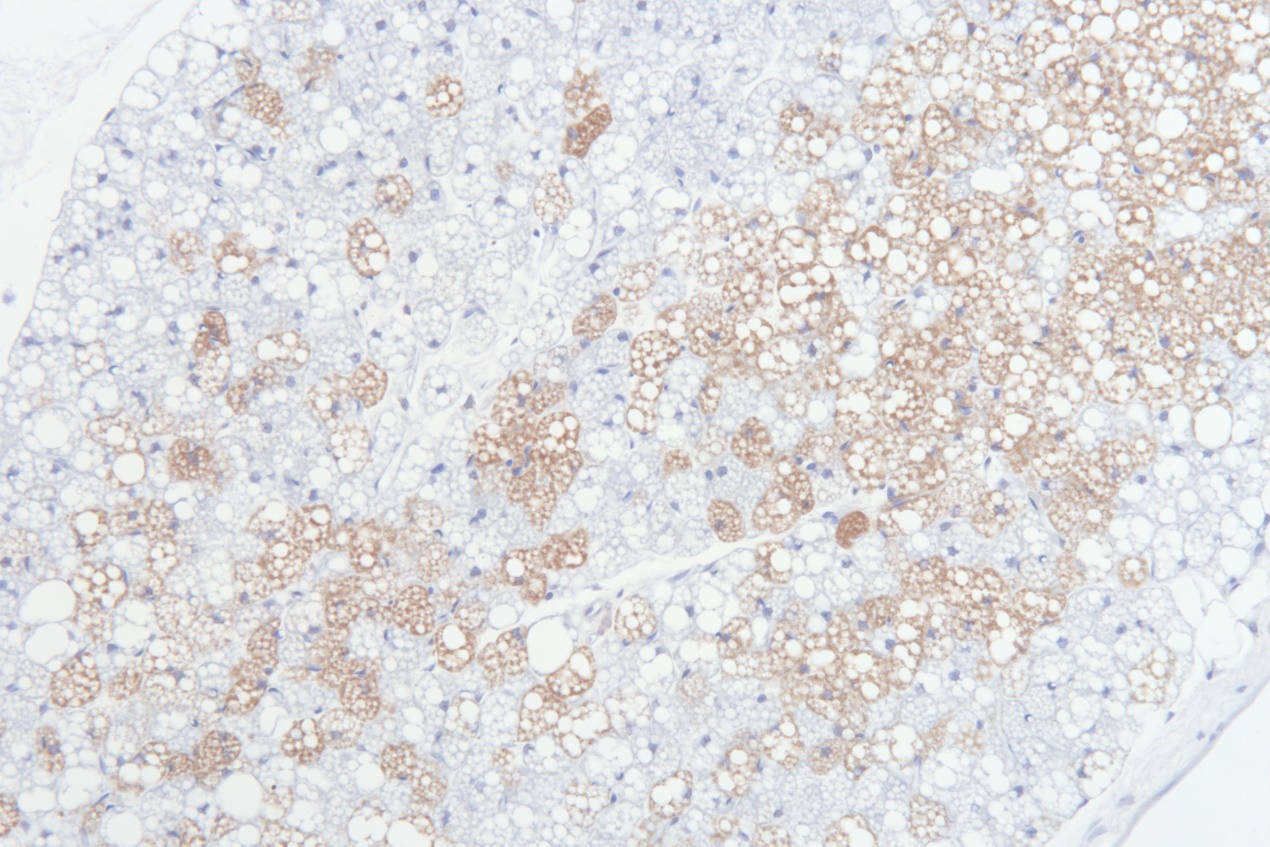


**Fig S9-9 OLZ+ AuNCs L**


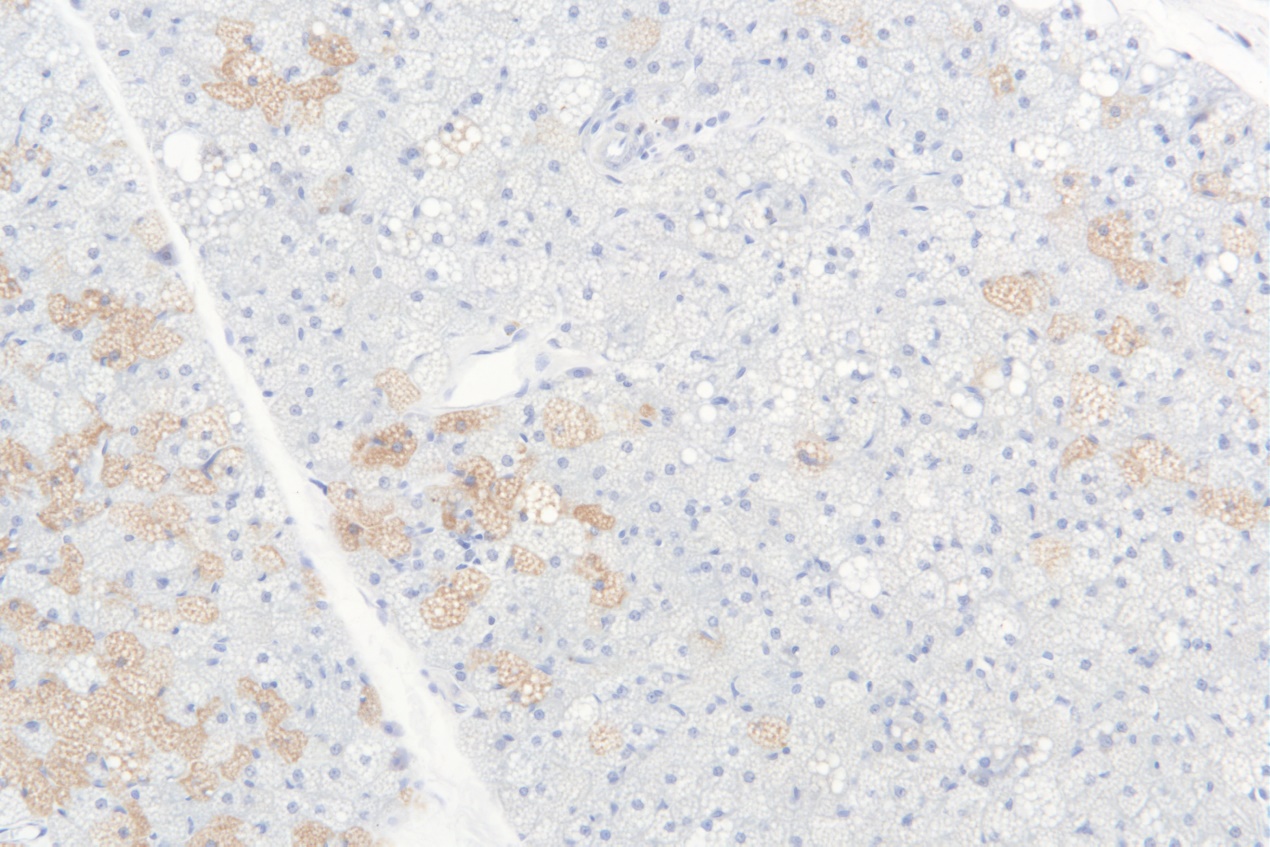


**Fig S9-10 AuNCs H**


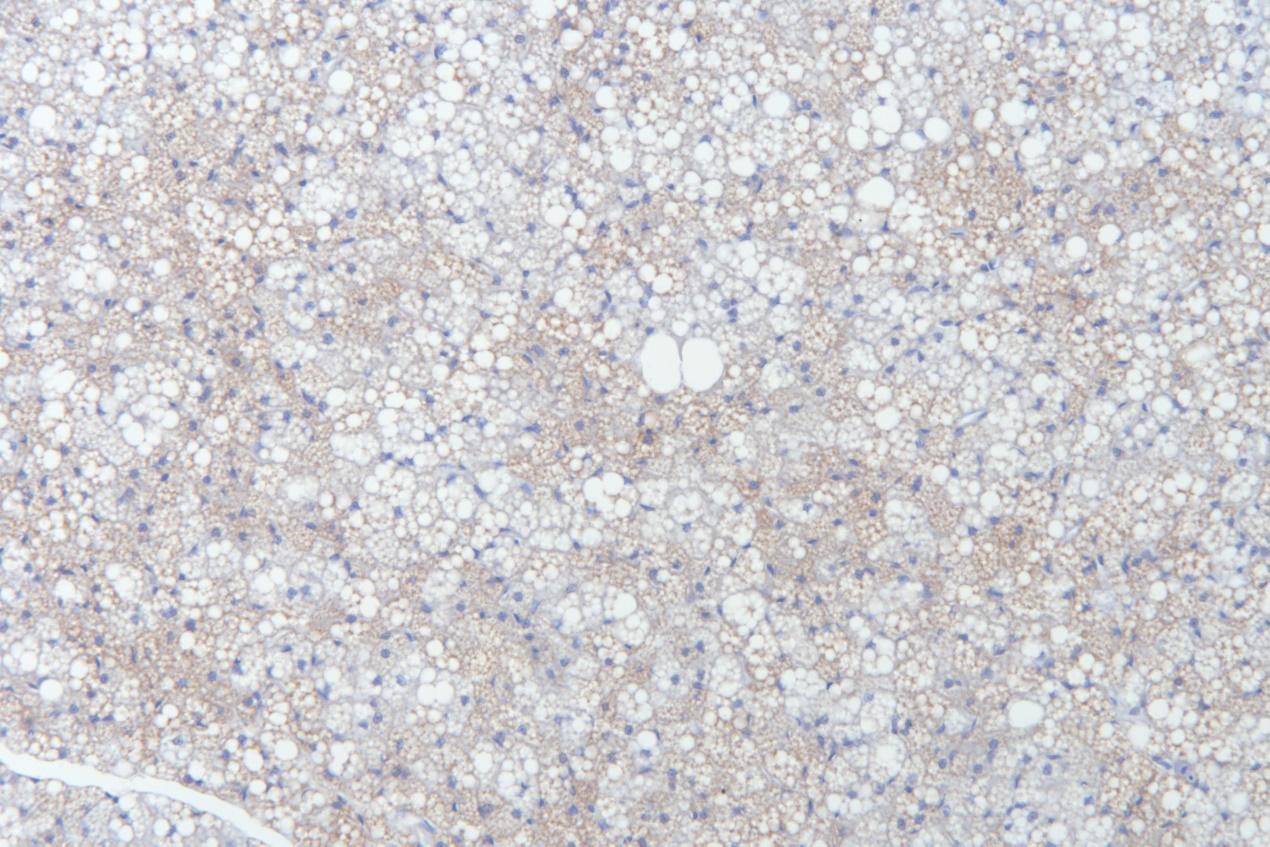


**Fig 4f. Immunohistochemistry of PGC-1α**

**Fig S9-11 CON**


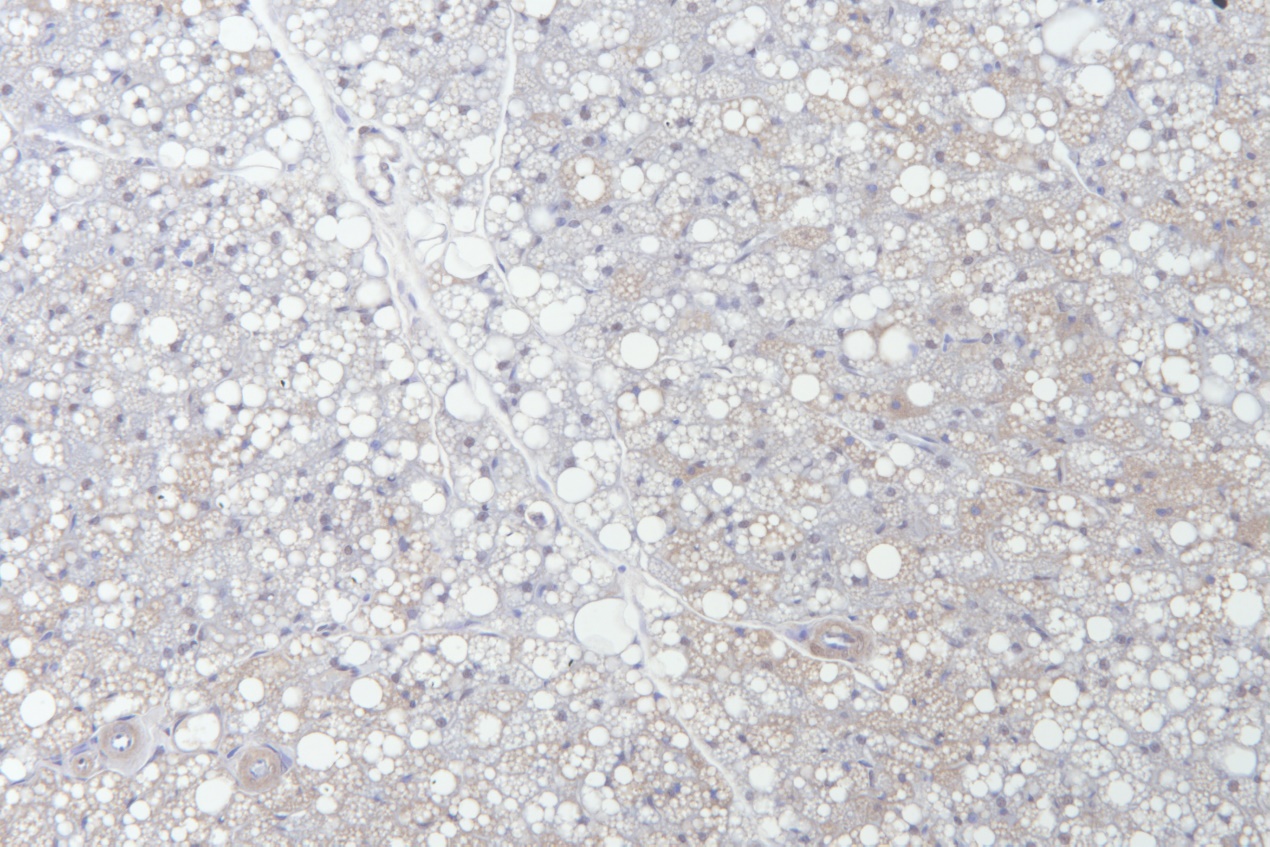


**Fig S9-12 OLZ**


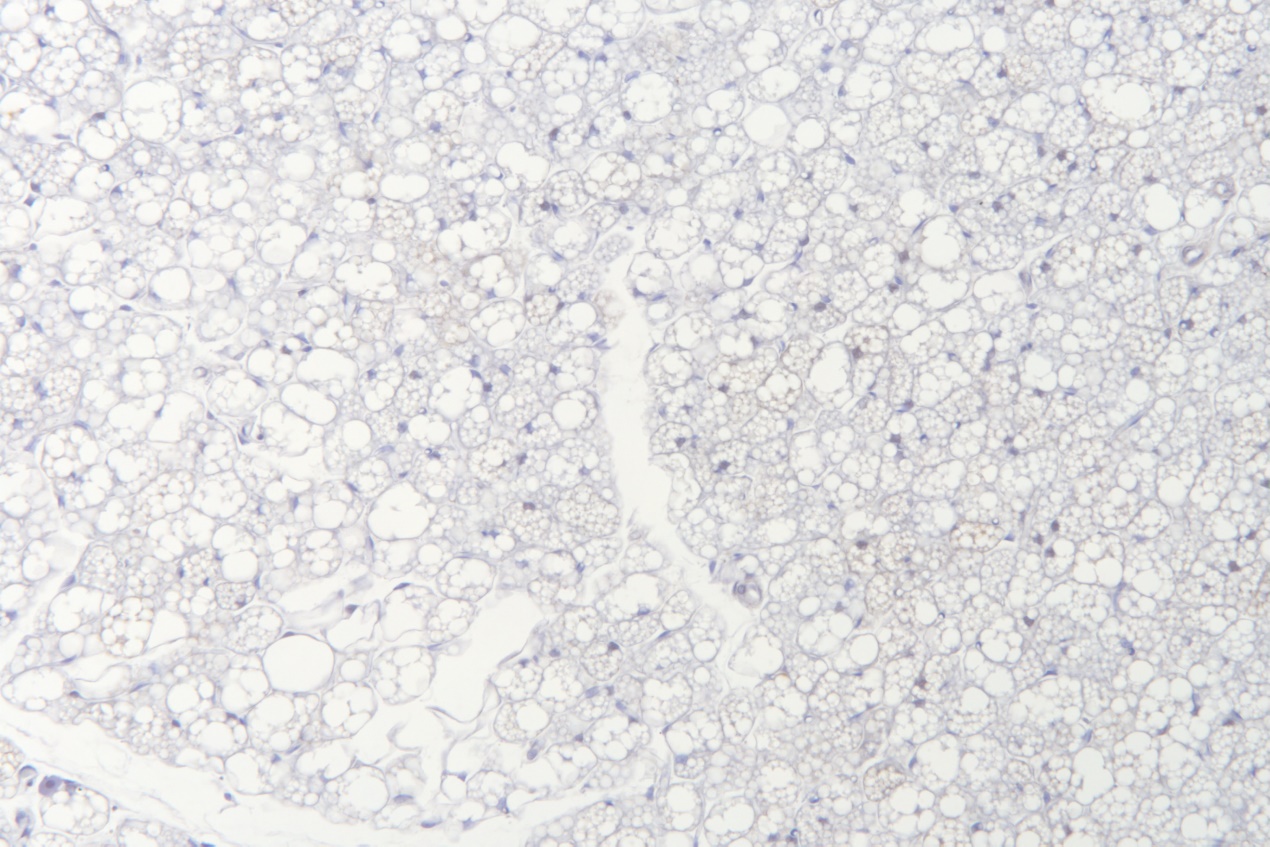


**Fig S9-13 OLZ+ AuNCs H**


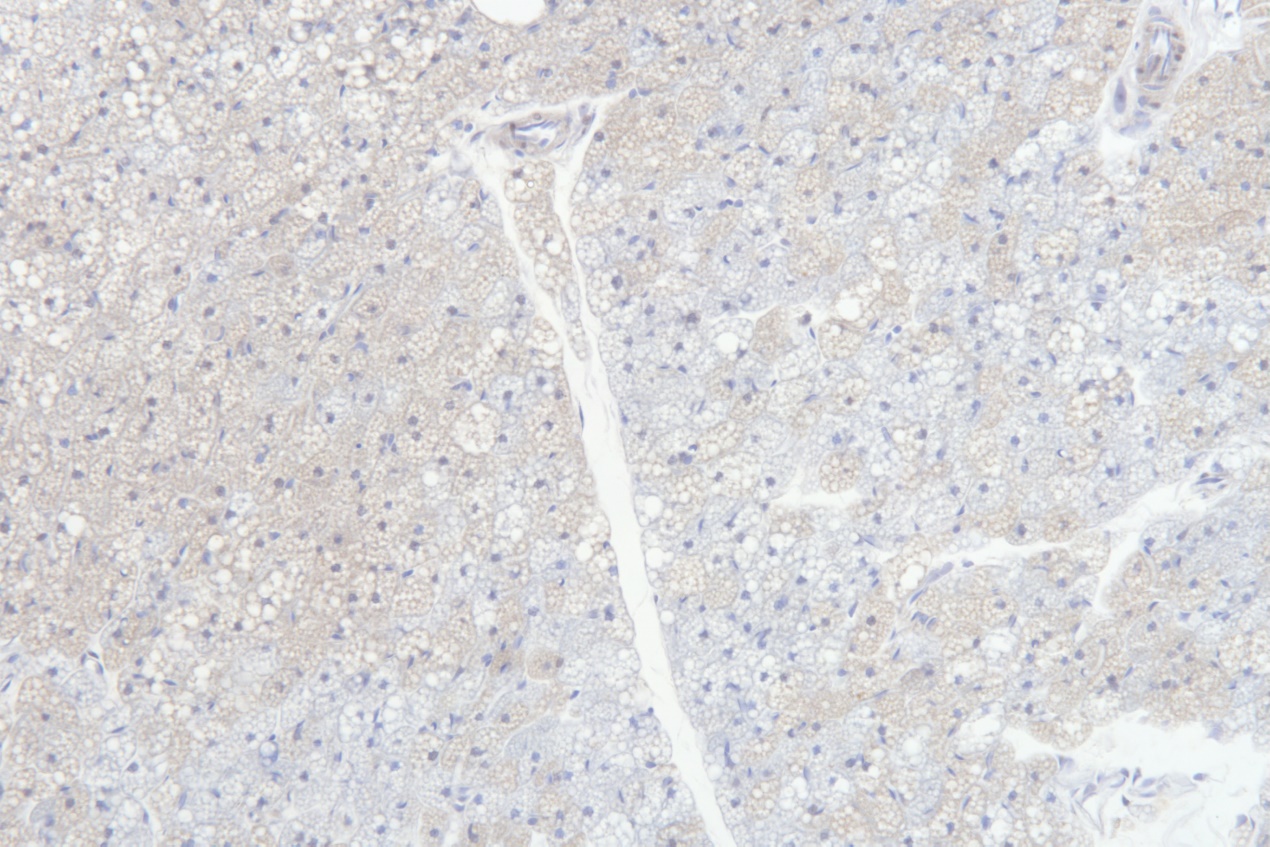


**Fig S9-14 OLZ+ AuNCs L**


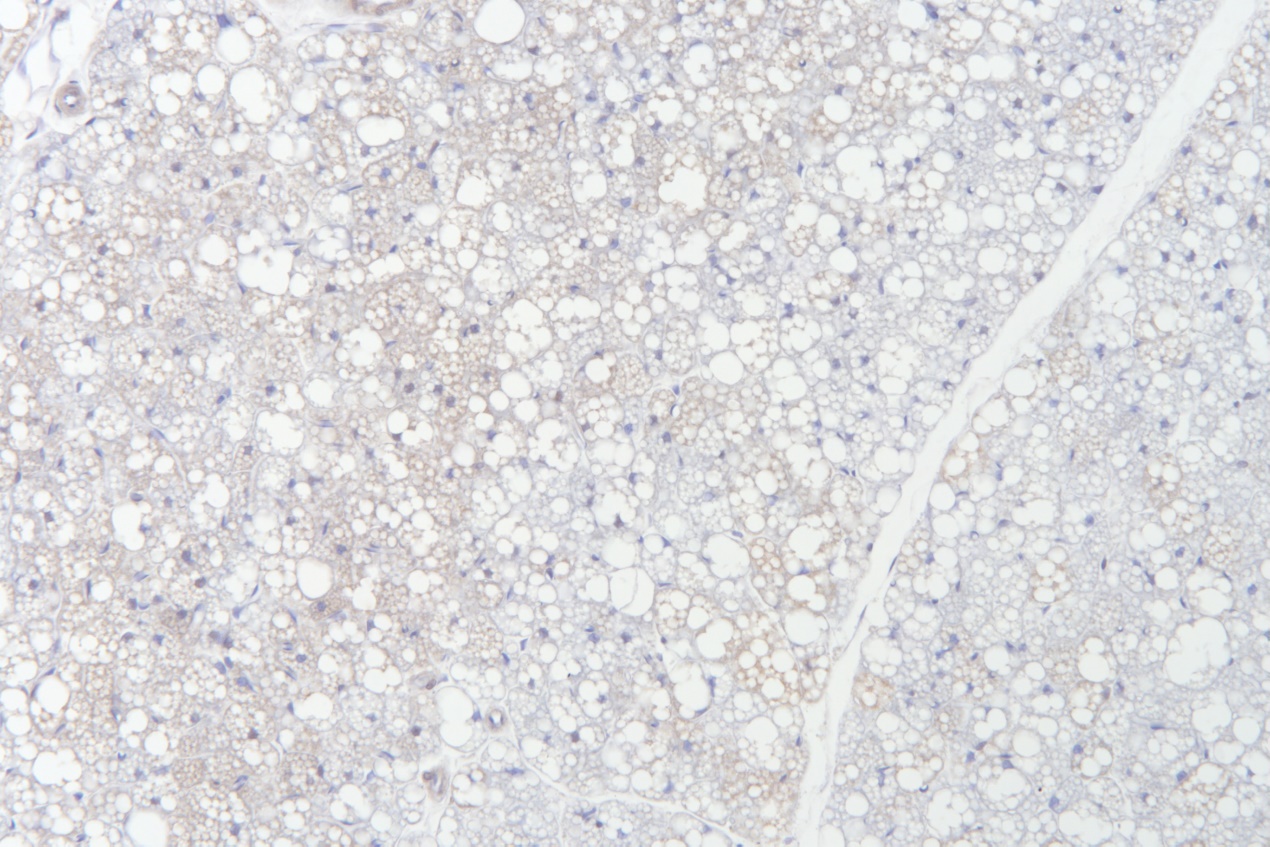


**Fig S9-15 AuNCs H**


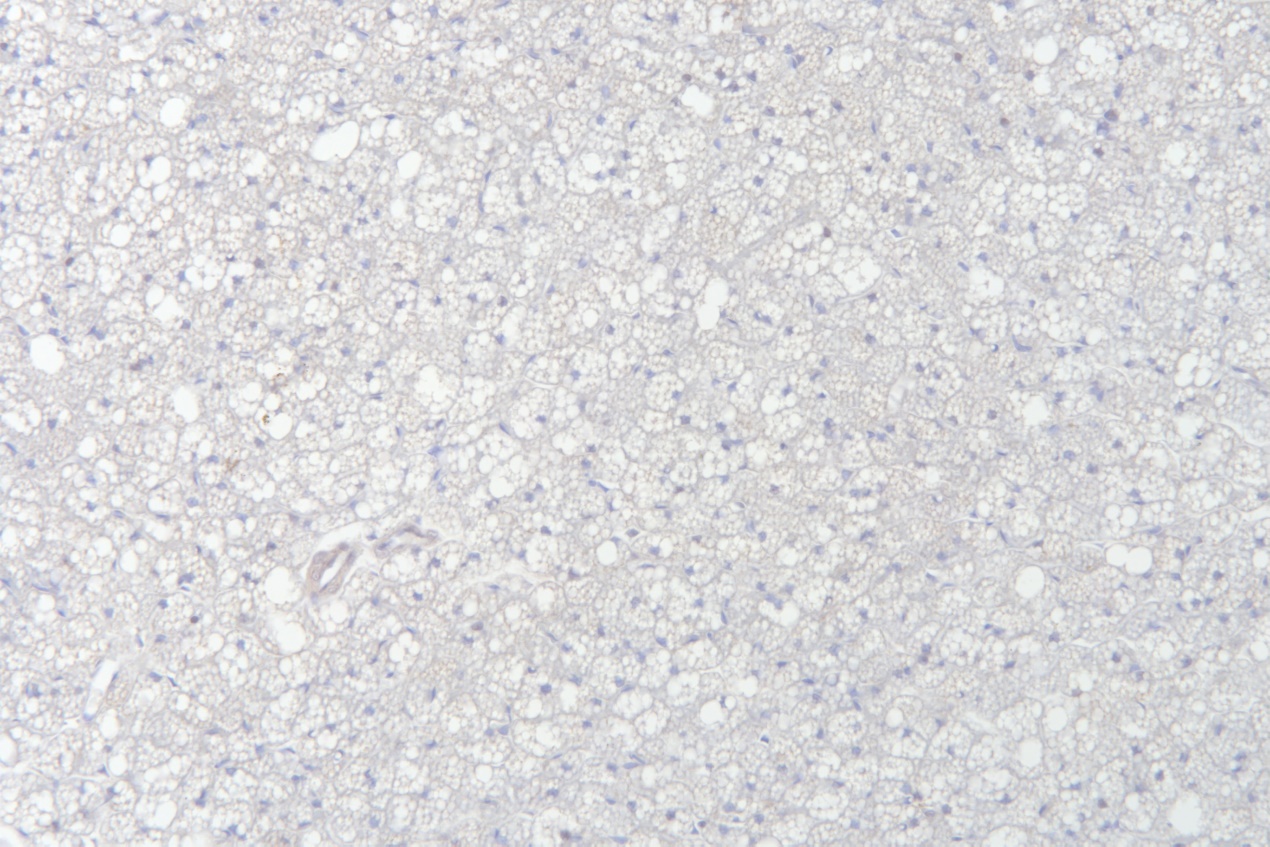


**Fig 4f. H&E staining of BAT**

**Fig S9-16 CON**

**
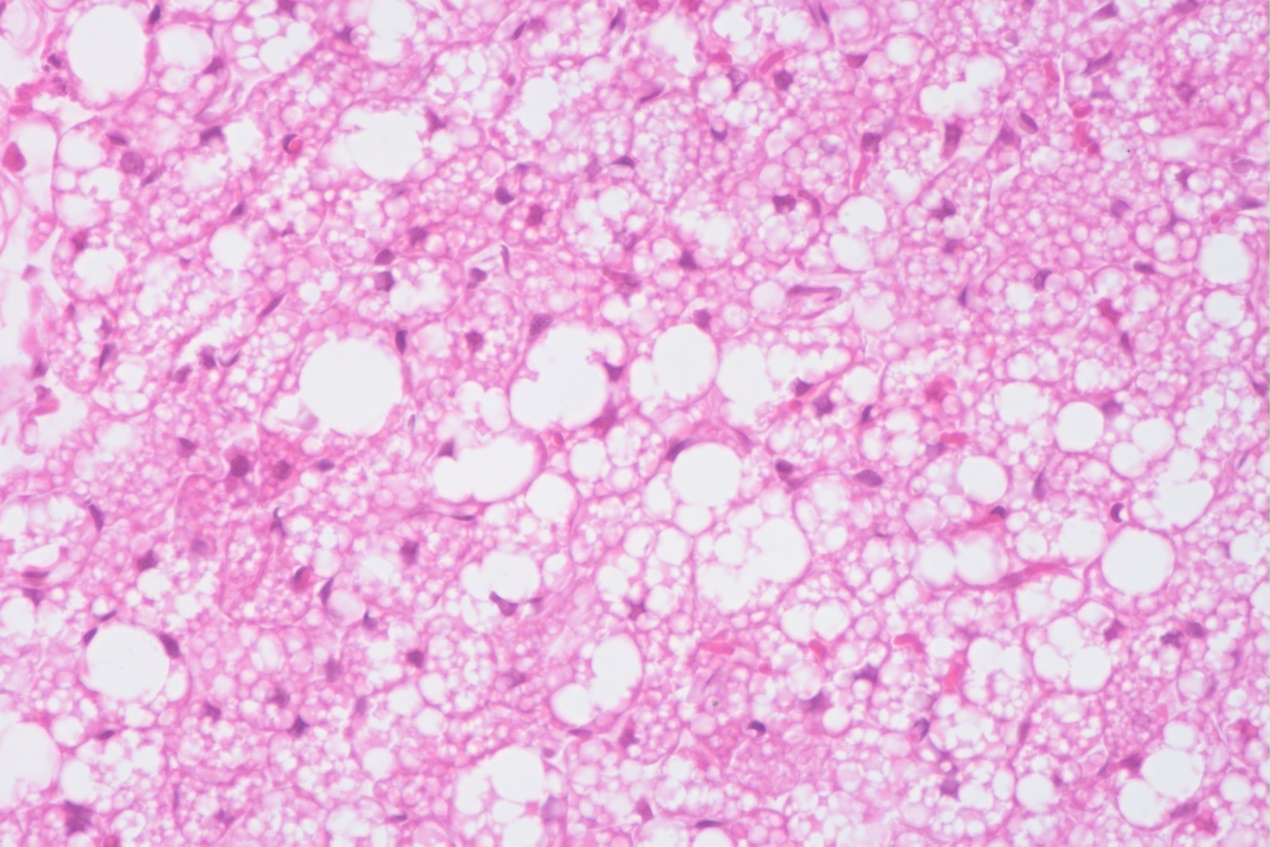
**

**Fig S9-17 OLZ**

**
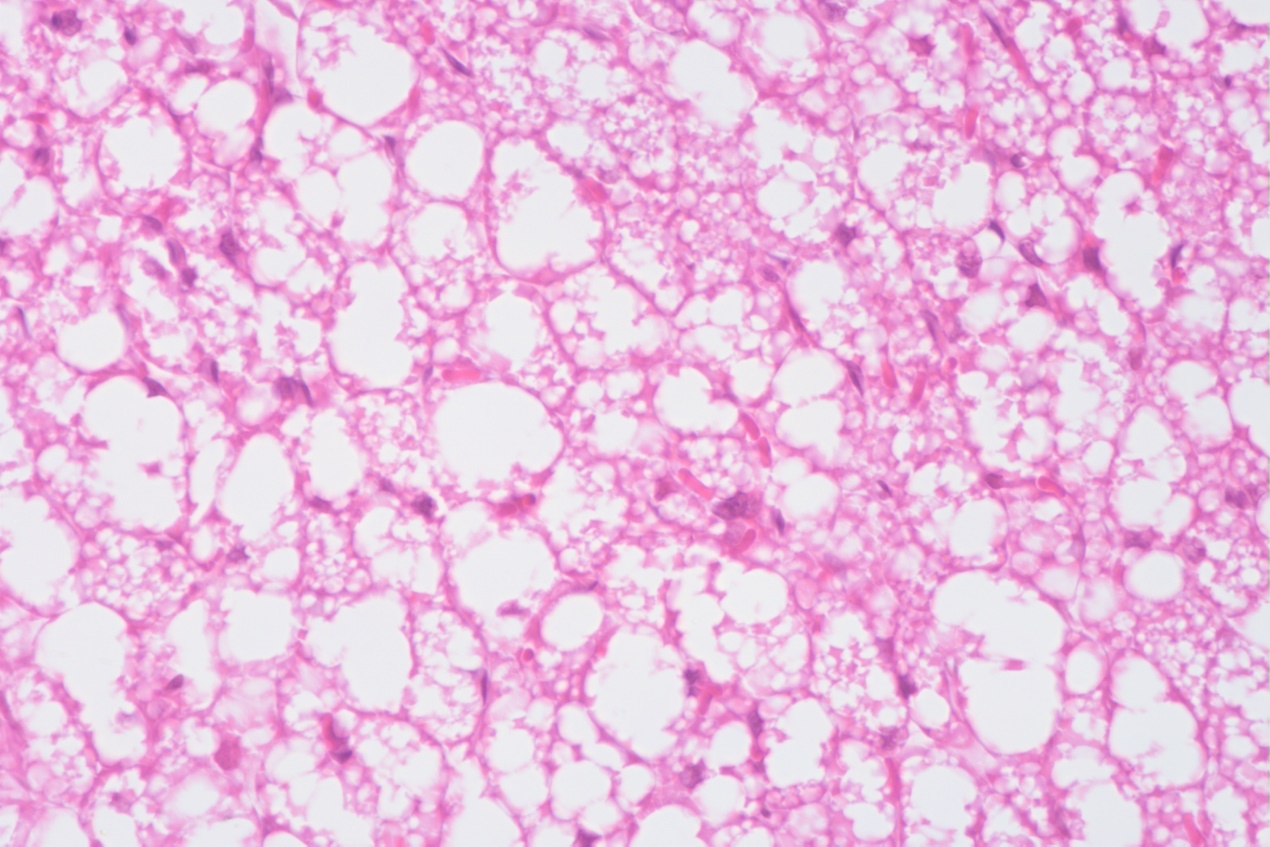
**

**Fig S9-18 OLZ+AuNCS H**

**
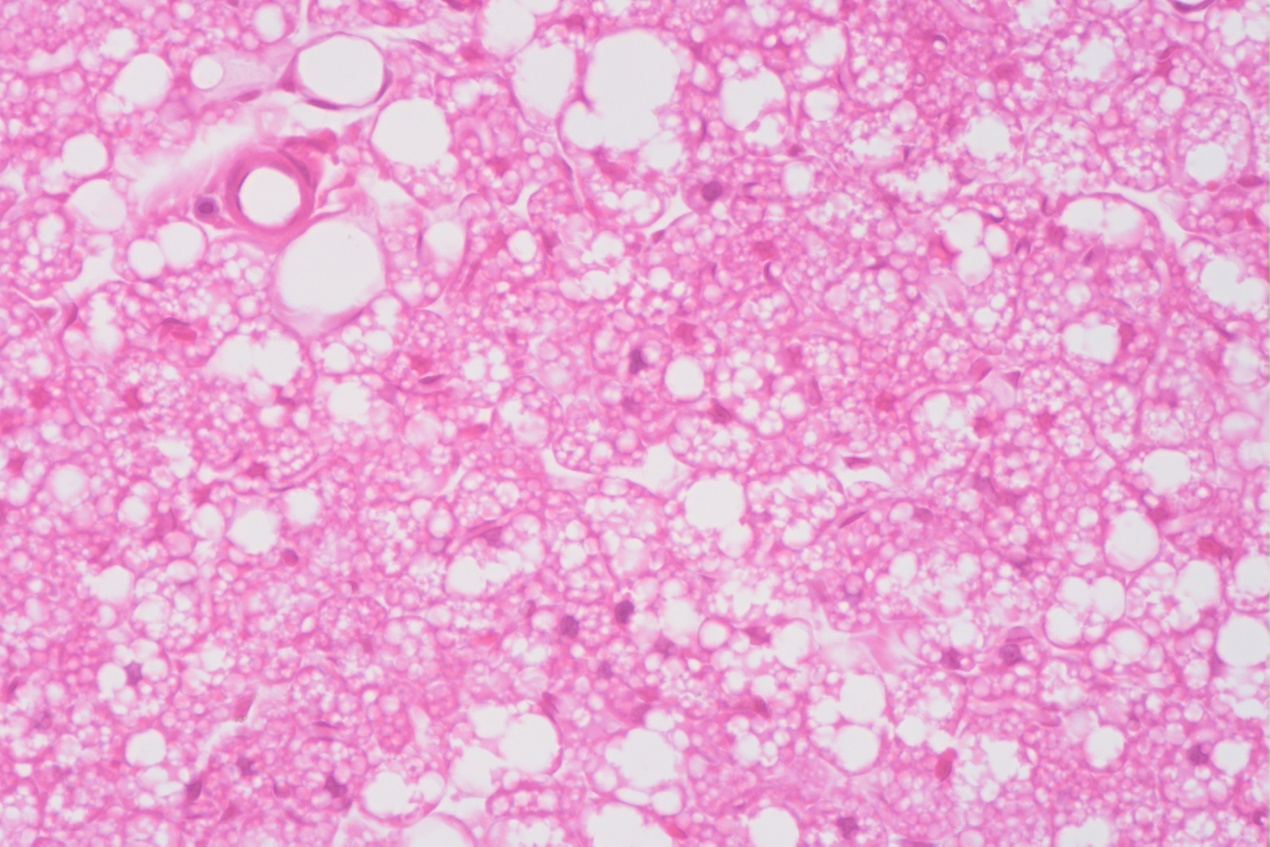
**

**Fig S9-19 OLZ+AuNCS L**

**
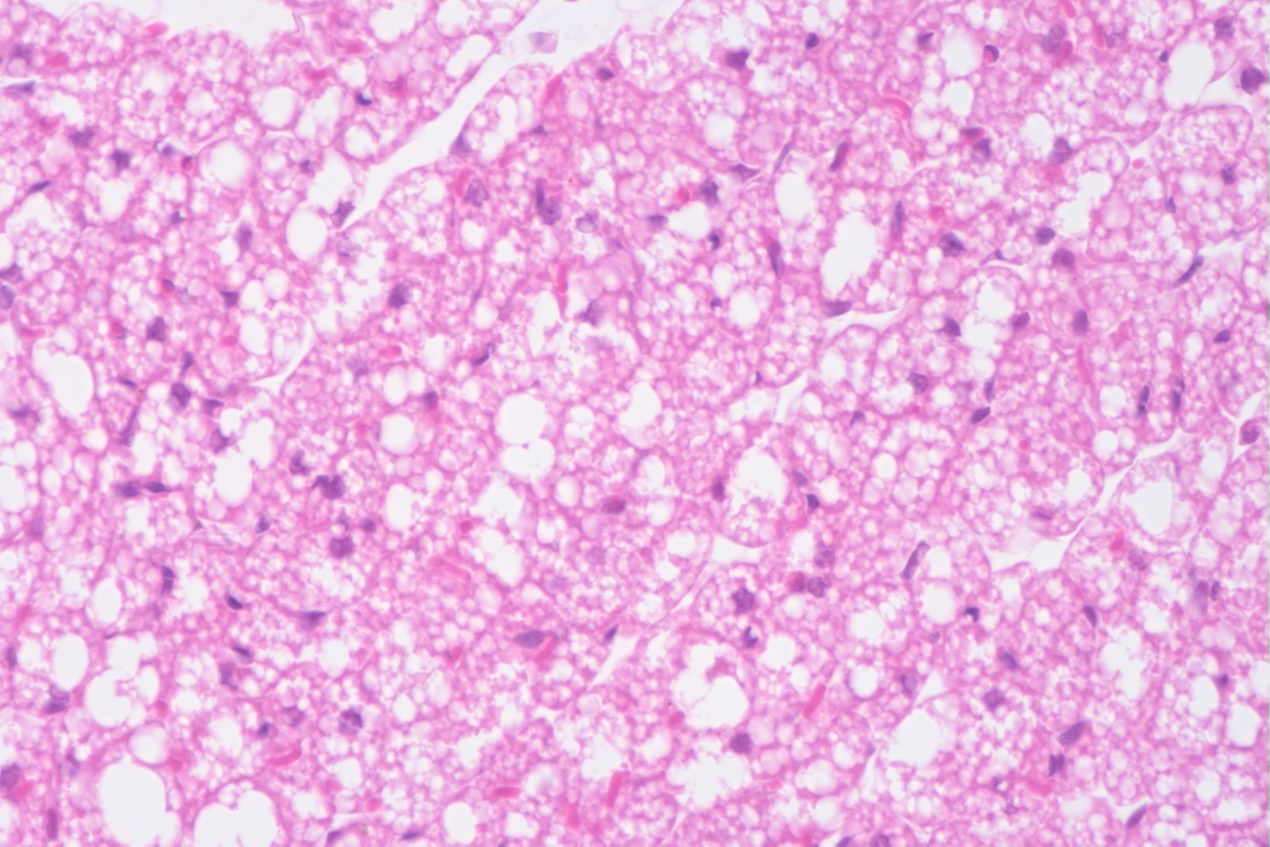
**

**Fig S9-20 AuNCs H**

**
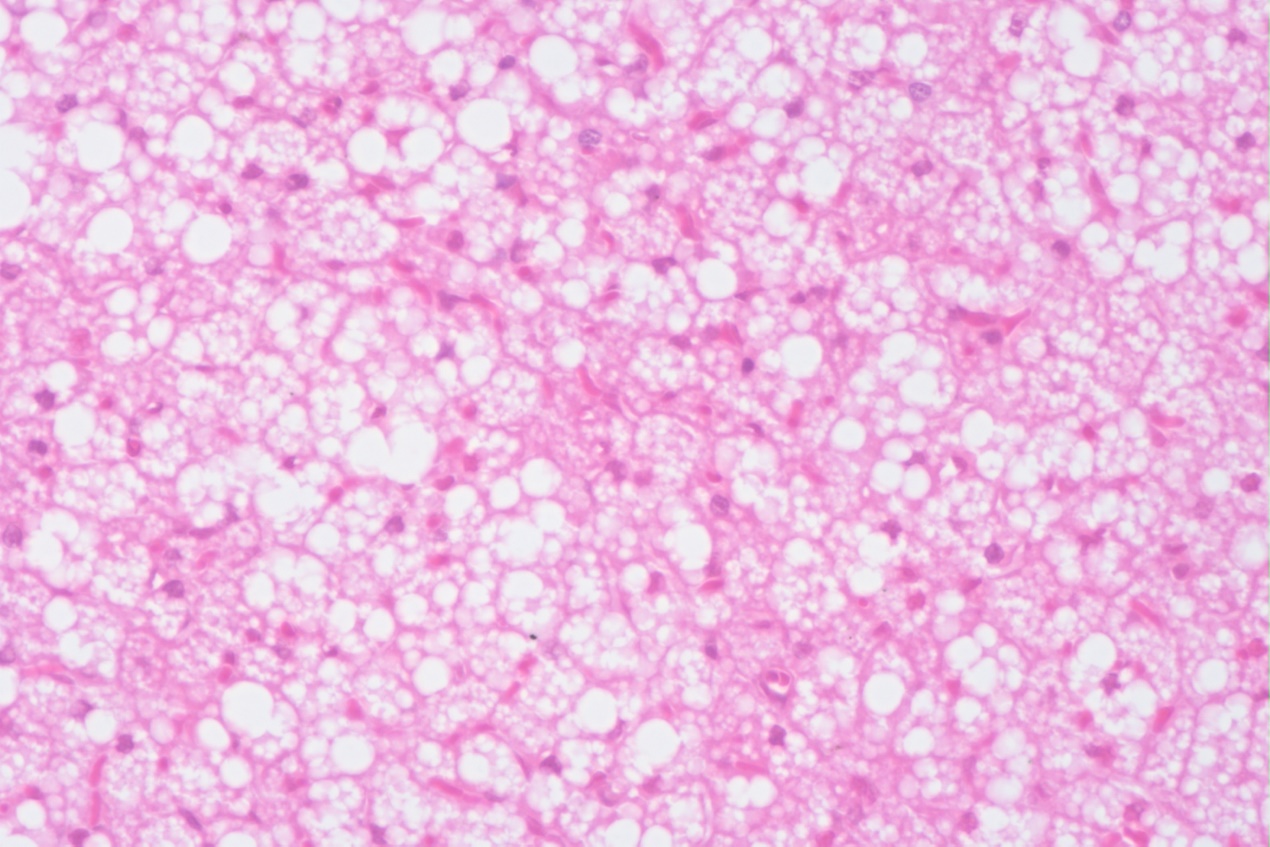
**

**5.** **Supplemental Statistical Section (main effects only)**

**Fig 2:**

**a:** Interaction between time and treatment; Day2, F(4,50)=1.202, *p*=0.322; Day4, F(4,50)=2.019, *p*=0.106; Day6, F(4,50)=2.07 *p*=0.099; Day8, F(4,50)=2.722, *p*=0.040; Day10, F(4,50)=1.923, *p*=0.121; Day12, F(4,50)=2.183, *p*=0.084; Day14, F(4,50)=4.043, *p*=0.006; Day16, F(4,50)=3.820, *p*=0.009; Day18, F(4,50)=4.300, *p*=0.005; two-way repeated measures ANOVA with within subjects factor of time and between subjects factor of treatment. Dunnett post hoc test

**b:** Interaction between time and treatment; Day1, F(4,50)=1.320, *p*=0.275; Day2, F(4,50)=1.837, *p*=0.136; Day3, F(4,50)=4.620, *p*=0.003; Day4, F(4,50)=11.773, *p*<0.0001; Day5, F(4,50)=6.701, *p*<0.0001; Day6, F(4,50)=10.377, *p*<0.0001; Day7, F(4,50)=6.458, *p*<0.0001; Day8, F(4,50)=5.385, *p*=0.001; Day9, F(4,50)=2.878, *p*=0.032; Day10, F(4,50)=2.308, *p*=0.071; Day11, F(4,50)=1.941, *p*=0.118; Day12, F(4,50)=0.969, *p*=0.433; Day13, F(4,50)=2.091, *p*=0.096; Day14, F(4,50)=1.704, *p*=0.164; Day15, F(4,50)=3.218, *p*=0.020; Day16, F(4,50)=2.095, *p*=0.095; Day17, F(4,50)=1.14, *p*=0.351; Day18, F(4,50)=4.639, *p*=0.003; two-way repeated measures ANOVA with within subjects factor of time and between subjects factor of treatment. Dunnett post hoc test

**c:** Interaction between time and treatment; Day2, t_(53)_=-0.339, *p*=0.736; Day4, t_(53)_=3.278, *p*=0.002; Day6, t_(53)_=4.825, *p*<0.0001; Day8, t_(53)_=4.988, *p*<0.0001; Day10, t_(53)_=4.508, *p*<0.0001; Day12, t_(53)_=5.498, *p*<0.0001; Day14, t_(53)_=3.886, *p*<0.0001; Day16, t_(53)_=3.891, *p*<0.0001; Day18, t_(53)_=2.807, *p*=0.007; Day20, t_(53)_=3.441, *p*=0.001; Day22, t_(53)_=4.212, *p*<0.0001; Day24, t_(53)_=4.088, *p*<0.0001; Day26, t_(53)_=3.851, *p*<0.0001; Day28, t_(53)_=3.841, *p*<0.0001; Student’s unpaired t-test. Day 30, F(4,50)=4.022, *p*=0.007; Day 32, F(4,50)=3.450, *p*=0.014; Day 34, F(4,50)=5.284, *p*=0.001; Day 36, F(4,50)=4.878, *p*=0.002; Day 38, F(4,50)=5.354, *p*=0.001; Day 40, F(4,50)=5.328, *p*=0.001; Day 42, F(4,50)=5.987, *p*=0.001; Day 44, F(4,50)=5.261, *p*=0.001; Day 46, F(4,50)=6.272, *p*<0.0001; Day 48, F(4,50)=7.163, *p*<0.0001; Day 50, F(4,50)=8.815, *p*<0.0001; Day 52, F(4,50)=7.105, *p*<0.0001; two-way repeated measures ANOVA with within subjects factor of time and between subjects factor of treatment. Dunnett post hoc test

**e:** Main effect of treatment; glucose at 0min: F(4,39)=4.427, *p*=0.005; 90min: F(4,38)=3.030, *p*=0.029, two-way ANOVA with Dunnett post hoc test. glucose at 30min,60min,120min, Mann-whitney U test

**f:** Main effect of treatment; Mann-whitney U test

**Fig 3.**

**c:** Main effect of treatment; F(4,15)=29.152, *p* < 0.0001; two-way ANOVA with Dunnett post hoc test

**d:** Main effect of treatment; F(4,15)=11.359, *p* < 0.0001; two-way ANOVA with Dunnett post hoc test

**e:** Main effect of treatment; F(4,15)=12.783, *p*< 0.0001; two-way ANOVA with Dunnett post hoc test

**k:** Main effect of treatment; F(4,15)=6.541, *p*= 0.003; two-way ANOVA with Dunnett post hoc test

**Fig 4.**

**b:** Main effect of treatment; F(4,15)=45.636, *p* < 0.0001; two-way ANOVA with Dunnett post hoc test

**c:** Main effect of treatment; F(4,15)=67.996, *p* < 0.0001; two-way ANOVA with Dunnett post hoc test

**d:** Main effect of treatment; F(4,15)=18.462, *p* < 0.0001; two-way ANOVA with Dunnett post hoc test

**e:** Main effect of treatment; F(4,15)=34.981, *p* < 0.0001; two-way ANOVA with Dunnett post hoc test

**g:** Main effect of treatment; Mann-whitney U test

**h:** Main effect of treatment; Mann-whitney U test

**i:** Main effect of treatment; F(4,15)=3.184, *p*=0.044; two-way ANOVA with Dunnett post hoc test

**Fig 5**

**b:** Main effect of treatment; F(4,41)=4.897, *p*=0.003; two-way ANOVA with Dunnett post hoc test

**Fig 6**

**b:** Main effect of treatment; F(2,12)=0.083, *p*=0.921; two-way ANOVA with Dunnett post hoc test

**c:** Main effect of treatment; F(2,12)=2.545, *p*=0.120; two-way ANOVA with Dunnett post hoc test

**d:** Main effect of treatment; F(2,12)=3.971, *p*=0.047; two-way ANOVA with Dunnett post hoc test

**e:** Main effect of treatment; F(2,12)=0.680, *p*=0.525; two-way ANOVA with Dunnett post hoc test

**f:** Main effect of treatment; F(2,12)=4.930, *p*=0.027; two-way ANOVA with Dunnett post hoc test

**g:** Main effect of treatment; F(2,12)=0.172, *p*=0.844; two-way ANOVA with Dunnett post hoc test

**h:** Main effect of treatment; F(2,12)=1.101, *p*=0.364; two-way ANOVA with Dunnett post hoc test

**i:** Main effect of treatment; F(2,12)=5.883, *p*=0.017; two-way ANOVA with Dunnett post hoc test

**j:** Main effect of treatment; Mann-whitney U test

**Fig S1**

**b:** Main effect of treatment; Mann-whitney U test

**c:** Main effect of treatment; H1R-24h, F(4,15)=12.866, *p* < 0.0001; two-way ANOVA with Dunnett post hoc test; pAMPK 24h, Mann-whitney U test

**Fig S2**

Main effect of treatment; perirenal: F(4,35)=0.568, *p*=0.688; periovary: F(4,35)=1.527, *p*=0.216; mesentary: F(4,34)=6.979, *p* < 0.0001; two-way ANOVA with Dunnett post hoc test

**Fig S3**

Main effect of treatment; triglycerides: F(4,35)=1.141, *p*=0.354; cholesterol: F(4,33)=0.335, *p*=0.853; two-way ANOVA with Dunnett post hoc test

**Fig S4**

**a:** Main effect of treatment; Mann-whitney U test

**b:** Main effect of treatment; Mann-whitney U test
